# The Adipokinetic Peptides in Diptera: Structure, Function, and Evolutionary Trends

**DOI:** 10.3389/fendo.2020.00153

**Published:** 2020-03-31

**Authors:** Gerd Gäde, Petr Šimek, Heather G. Marco

**Affiliations:** ^1^Department of Biological Sciences, University of Cape Town, Cape Town, South Africa; ^2^Biology Centre, Czech Academy of Sciences, Ceské Budejovice, Czechia

**Keywords:** diptera, adipokinetic peptides, mass spectrometry, adipokinetic and hypertrahalosemic biological assays, fly phylogeny

## Abstract

Nineteen species of various families of the order Diptera and one species from the order Mecoptera are investigated with mass spectrometry for the presence and primary structure of putative adipokinetic hormones (AKHs). Additionally, the peptide structure of putative AKHs in other Diptera are deduced from data mining of publicly available genomic or transcriptomic data. The study aims to demonstrate the structural biodiversity of AKHs in this insect order and also possible evolutionary trends. Sequence analysis of AKHs is achieved by liquid chromatography coupled to mass spectrometry. The corpora cardiaca of almost all dipteran species contain AKH octapeptides, a decapeptide is an exception found only in one species. In general, the dipteran AKHs are order-specific- they are not found in any other insect order with two exceptions only. Four novel AKHs are revealed by mass spectrometry: two in the basal infraorder of Tipulomorpha and two in the brachyceran family Syrphidae. Data mining revealed another four novel AKHs: one in various species of the infraorder Culicumorpha, one in the brachyceran superfamily Asiloidea, one in the family Diopsidae and in a Drosophilidae species, and the last of the novel AKHs is found in yet another *Drosophila*. In general, there is quite a biodiversity in the lower Diptera, whereas the majority of the cyclorraphan Brachycera produce the octapeptide Phote-HrTH. A hypothetical molecular peptide evolution of dipteran AKHs is suggested to start with an ancestral AKH, such as Glomo-AKH, from which all other AKHs in Diptera to date can evolve via point mutation of one of the base triplets, with one exception.

## Introduction

There are surely a few facts about the importance of Diptera for ecology, medicine and agriculture that are not so well-known to the general public and non-dipteran specialists alike. Most associations with “flies” in general are about the role of mosquitoes as vectors of terribly infectious diseases, such as malaria or yellow fever, or about the use of the vinegar fly *Drosophila melanogaster* as model organism for genetic experiments into development, aging, behavior, metabolism and diseases. Mosquitoes (Family: Culicidae) are widely known as “detrimental” dipteran species, for they are vectors for Dengue-, West Nile- Zika- and yellow fever, as well as for malaria and encephalitis (https://www.who.int/neglected_diseases/vector_ecology/mosquito-borne-diseases/en/). Certain house flies (Family: Muscidae) and blow flies (Family: Calliphoridae) transmit disease microorganisms that lead to, e.g., cholera, dysentery and gastroenteritis in humans (1), and from horse flies (Family: Tabanidae), diseases may be effected in other animals, such as equine infectious anemia, and anthrax in cattle and sheep (2). Next to such detrimental effectors, it is perhaps understandable that many people overlook the fact that there are also beneficial fly species, e.g., hover flies (Family: Syrphidae); these are the second most important pollinators after the Hymenoptera (3). Additionally, there are specialist pollinators known from other dipteran families, such as the long-tongued horse flies (Family: Tabanidae) or the cocoa-tree pollinator midges (Family: Ceratopogonidae) (3). Other benign functions are fulfilled by certain fly larvae of the family Calliphoridae, used for medicinal purposes to clean wounds by consuming necrotic tissues, and as producers of pharmacologically active substances such as antimicrobial, antiviral, and antitumor compounds (4). Also, fly larvae of the black soldier fly *Hermetia illucens* (Family: Stratiomyidae), feed voraciously on household and agricultural waste products. These insect decomposers are now reared at industrial scale, and their pupae used for the economic production of oils and dried protein feed for chicken and fish (5). Finally, in forensic entomology the succession order of larvae of various flies on/in the decomposing human corpse is indicative of the time of death (6) and provides yet another example of the positive usefulness of Diptera.

From the above remarks it should be clear that “the flies” are quite a diverse order with respect to being classified as “pest” or “beneficial.” This is not surprising when considering the vast number of fly species: the order Diptera comprises about 152 000 described species (7). Until recently the major divide has been (a) the Nematocera [mostly crane flies (Family: Tipulidae) and mosquitoes, characterized by long antennae] and (b) Brachycera which harbor *inter alia* the above-mentioned families—Tabanidae, Drosophilidae and Muscidae—which are all characterized by short antennae. The newest phylogenomic research takes 149 out of 157 families of Diptera into account and interprets molecular data from 14 nuclear loci and from many complete mitochondrial genomes, as well as 371 morphological characters (8). The comprehensive study presented a phylogenetic tree that shows (i) the monophyly of Diptera with Mecoptera (scorpion flies) and Siphonaptera (fleas) as closest relatives; (ii) paraphyly in the former Nematocera which are now rather called lower Diptera; the rare and species-poor families Deuterophlebiidae (mountain midges) and Nymphomyiidae are at the base of the fly tree, followed by the lower dipteran infraorders Tipulomorpha and Culicomorpha; (iii) the clade Neodiptera comprising of the infraorder Bibionomorpha (marsh flies and gall midges) is the sister group of Brachycera; (iv) the major clades of Brachycera are the Orthorrapha [Tabanomorpha, Stratiomorpha (such as soldier flies) and Asiloidea (such as robber flies)], the Schizophora in the Cyclorrapha [Tetritoidea (fruit flies) and Ephydroidea (relatives of *Drosophila*)] and the Calyptrate inside the Cyclorrapha (such as tsetse flies, house flies and blow flies)] (8).

In the current study, we determine the primary structure of an important metabolic hormone from the so-called adipokinetic hormone (AKH) family in major families of lower Diptera and Brachycera with the aim to establish how the biodiversity and distribution of various AKH sequences follows the afore-mentioned phylogeny. Previously, we have successfully implemented the use of the primary structure of AKHs in various insect orders to verify certain phylogenetic trends and ancestral relationships [e.g., (9–13)]. The AKH is one of many biologically active neuropeptides in insects. Its major function is regulation of metabolism and it can be compared in a functional respect with the vertebrate hormone, glucagon. Structurally, however, the AKH peptide and its G-protein coupled receptor is related to the vertebrate gonadotropin releasing hormone (GnRH) system, and it is suggested to form a large peptide superfamily together with two other insect neuropeptide systems: corazonin, and adipokinetic hormone/corazonin-related peptide (14–16). AKHs are synthesized and released from the corpus cardiacum (CC), a neurohemal organ. The peptides are characterized by a chain length of 8 to 10 amino acids and post-translationally modified N- and C-termini (pyroglutamate and carboxyamidation, respectively). At position two from the amino terminal, AKHs have either an aliphatic amino acid (leucine, isoleucine, or valine) or an aromatic amino acid (phenylalanine); position three is either a threonine or asparagine residue; at position four one finds either the aromatic phenylalanine or tyrosine; position five has either threonine or serine; position eight is always the aromatic tryptophan and position nine comprises of a simple glycine, whereas at position six, seven, and ten there is a large variability of amino acids possible (17, 18).

The first two AKH peptides of Diptera fully known by primary structure were isolated from the CC of the horse fly *Tabanus atratus* (Family: Tabanidae) and had the sequence of an octapeptide (pELTFTPGW amide) and a decapeptide [pELTFTPGWGY amide; thus, the octapeptide extended by two amino acids, (19)]. The code-names for these peptides were derived from their biological activity ascertained in heterologous biological assays: Tabat-AKH for the octapeptide and Tabat-HoTH for the decapeptide because these peptides, respectively, increased the lipids in the hemolymph (adipokinetic effect) or decreased the concentration of trehalose (hypotrehalosemic effect) in the hemolymph of the face fly *Musca autumnalis* (dipteran Family: Muscidae) (19). Homologous bioassays in the horse fly *Tabanus lineolata* set the record straight and came up with some strikingly different results: the octapeptide, which was called an “adipokinetic” peptide, had no hyperlipemic effect and caused only a slight increase of carbohydrates in the hemolymph of *T. lineolata*, whereas the decapeptide, called a “hypotrehalosemic peptide,” was a very effective adipokinetic and hyperglycemic hormone (20). Regardless of the specific action of these peptides in different insect species, they are generically known as members of the adipokinetic hormone family, in short AKHs.

The next dipteran AKH was elucidated a year after the tabanid peptides, isolated and sequenced from CC of the blowfly *Phormia* (=*Protophormia*) *terraenovae* (Family: Calliphoridae) as an octapeptide [pELTFSPDW amide; (21)] that regulates carbohydrate metabolism and is released during induced stress (22) and was, hence, code-named Phote-HrTH. This was the first member of the AKH family that had a charged amino acid; all other AKHs up to then were neutral molecules. A peptide with the same sequence was found at the same time in the vinegar fly *D*. *melanogaster* [Family: Drosophilidae; (23)], where biological assays established its activity as cardioacceleratory in prepupae of *D*. *melanogaster* (24). Phote-HrTH has subsequently been identified in a number of Dipteran species (including in larval specimens of *Drosophila melanogaster*, see Table 1) and is also known for its modulatory action on specific crop muscles of *Phormia regina* (44) that enables delivery of ingested food from the crop to the midgut for digestion. In the post-genomic era, *D. melanogaster* has become the established model insect for research on energy metabolism at the molecular level, with engineered AKH mutants, transgenic insect lines with specific modified cellular components (e.g., transcription factors, ATP-sensitive K^+^ channels, cGMP-dependent protein kinase), ablated endocrine cells, nutrient stress and other parameters, providing information to elucidate the pathways involved in glucose and lipid homeostasis, as well as disease states such as obesity, diabetes and aging [see recent reviews by Gáliková and Klepsatel (45) and Marco and Gäde (18)].

**Table 1:** The distribution of AKH peptides in the Diptera, to date: primary sequence and calculated protonated mass.

**Higher taxonomy**	**Family**	**Species**	**AKH name**	AKH Sequence^*^	**[M+H]^**+**^**	References^**^
Lower Diptera, Tipulomorpha	Tipulidae	*Tipula paludosa*	Glomo-AKH	pELTFSPGWa	917.4516	This study
			Tippa-CC-I	pELTYSPSWa	963.4571	
			Tippa-CC-II	pELTFSPSWa	947.4621	
		*Tipula oleracea*	Tippa-CC-II	pELTFSPSWa	947.4621	JXPP01112122.1
		Tipula oleracea?^***^	Glomo-AKH	pELTFSPGWa	917.4516	This study
			Tippa-CC-I	pELTYSPSWa	963.4571	
			Tippa-CC-II	pELTFSPSWa	947.4621	
Lower Diptera, Psychodomorpha	Psychodidae	*Lutzomyia longipalpis*	Tabat-AKH	pELTFTPGWa	931.4672	JH689332.1
		*Phlebotomus papatasi*	Tabat-AKH	pELTFTPGWa	931.4672	A0A1B0DLM1
Lower Diptera, Culicumorpha	Chironomidae	*Clunio marinus*	Aedae-AKH	pELTFTPSWa	961.4778	CRK93994.1
Lower Diptera, Culicumorpha	Culicidae	*Culex quinquefasciatus/ pipiens*	Aedae-AKH	pELTFTPSWa	961.4778	(25); B0W1A8
		*Aedes aegypti*	Aedae-AKH	pELTFTPSWa	961.4778	(25, 26); AAEL011996-PA
		*Aedes albopictus*	Aedae-AKH	pELTFTPSWa	961.4778	XP_029714753.1
		*Anopheles gambiae*	Anoga-HrTH	pELTFTPAWa	945.4829	(27); A0NGF4
		15 Other Anopheles species^a^	Anoga-HrTH	pELTFTPAWa	945.4829	
		*Anopheles dirus complex*	NOVEL 1	pELTFTPTWa	975.4934	A0A182NZ26
		*Anopheles farauti complex*	NOVEL 1	pELTFTPTWa	975.4934	A0A182R1D8
Lower Diptera, Culicumorpha	Chaoboridae	*Chaoborus trivitattus*	NOVEL 1	pELTFTPTWa	975.4934	JXOU01007118.1
		*Mochlonyx cinctipes*	NOVEL 1	pELTFTPTWa	975.4934	JXPH01036237.1
Brachycera, Orthorrapha, Tabanomorpha	Tabanidae	*Tabanus atratus*	Tabat-AKH Tabat-HoTH	pELTFTPGWa pELTFTPGWGYa	931.4672 1151.5520	(19); P14595.2 & P14596.2
Brachycera, Orthorrapha, Stratiomyomorpha	Stratiomyidae	*Hermetia illucens*	Tabat-AKH	pELTFTPGWa	931.4672	This study
		*Hermetia illucens*		pELTFTGQWa	962.4730	(28); JXPW01153780.1
Brachycera, Orthorrapha, Asiloidea	Asilidae	*Dasypogon diadema*	NOVEL 2	pELTFTPVWa	973.5142	QYTT01077274.1
Brachycera, Cyclorrapha, - Syrphoidea	Syrphidae	*Volucella pellucens*	Glomo-AKH	pELTFSPGWa	917.4516	This study
			Phote-HrTH	pELTFSPDWa	975.4571	
			Volpe-CC	pELTFSPYWa	1023.4934	
		*Volucella zonaria*	Glomo-AKH	pELTFSPGWa	917.4516	This study
			Phote-HrTH	pELTFSPDWa	975.4571	
			Volpe-CC	pELTFSPYWa	1023.4934	
		*Chrysotoxum cautum*	Glomo-AKH	pELTFSPGWa	917.4516	This study
			Volpe-CC	pELTFSPYWa	1023.4934	
		*Allograpta fuscotibialis*	Volpe-CC	pELTFSPYWa	1023.4934	This study
		*Eristalis ssp* mixture	Eriss-CC	pELTFSAGWa	891.4359	This study
			Glomo-AKH	pELTFSPGWa	917.4516	
			Phote-HrTH	pELTFSPDWa	975.4571	
			Volpe-CC	pELTFSPYWa	1023.4934	
		*Eristalis tenax*	Glomo-AKH	pELTFSPGWa	917.4516	This study
			Volpe-CC	pELTFSPYWa	1023.4934	
		*Eristalis ssp* 1 (not *E. tenax*)	Glomo-AKH Volpe-CC	pELTFSPGWa pELTFSPYWa	917.4516 1023.4934	This study
		*Eristalis ssp* 2 (not *E. tenax*)	Eriss-CC Volpe-CC	pELTFSAGWa pELTFSPYWa	891.4359 1023.4934	This study
		*Eristalis dimidiate*	Glomo-AKH	pELTFSPGWa	917.4516	JXPC01087303.1
Brachycera, Cyclorrapha, Schizophora	Sepsidae	*Themira minor*	Phote-HrTH	pELTFSPDWa	975.4571	JXPZ01029543.1
Brachycera, Cyclorrapha, Schizophora, Tephritoidea	Tephritidae	*Ceratitis capitata*	Phote-HrTH	pELTFSPDWa	975.4571	This study; XP_004526727.1
		*Batrocera dorsalis*	Phote-HrTH	pELTFSPDWa	975.4571	XP_011211021.1
		*Batrocera oleae*	Phote-HrTH	pELTFSPDWa	975.4571	XP_014099190.1
		*Batrocera latifrons*	Phote-HrTH	pELTFSPDWa	975.4571	XP_018793135.1
		*Zeugodacus cucurbitae*	Phote-HrTH	pELTFSPDWa	975.4571	XP_011184633.1
		*Rhagoletis zephyra*	Phote-HrTH	pELTFSPDWa	975.4571	XP_017484104.1
		*Rhagoletis cerasi*	Phote-HrTH	pELTFSPDWa	975.4571	(29)
Brachycera, Cyclorrapha, Schizophora	Diopsidae	*Sphyracephala brevicornis* *Teleopsis dalmanni*	NOVEL 3 NOVEL 3	pELTFSPNWa pELTFSPNWa	*974.4730* 974.4730	JXPL01104182.1; (28) NLCU01016345.1; (28)
Brachycera, Cyclorrapha, Schizophora	Agromyzidae	*Liriomyza trifolii*	Phote-HrTH	pELTFSPDWa	975.4571	JXHJ01005265.1
Brachycera, Cyclorrapha, Schizophora, Ephydroidea	Ephydridae	*Cirrula hians*	Phote-HrTH	pELTFSPDWa	975.4571	JXOS01046117.1
Brachycera, Cyclorrapha, Schizophora Ephydroidea	Drosophilidae	*Drosophila melanogaster*	Phote-HrTH	pELTFSPDWa	975.4571	(23, 30–33); NP_523918.1
Brachycera, Cyclorrapha, Schizophora Ephydroidea	Drosophilidae	17 Other *Drosophila* species^b^	Phote-HrTH	pELTFSPDWa	975.4571	(34, 35); and data bases
		*Drosophila grimshawi*	NOVEL 3	pELTFSPNWa	974.4730	(34); XP_001983486.1
		*Drosophila ficusphila*	NOVEL 4	pELTYSPDWa	991.4520	XP_017059889.1
		*Scaptodrosophila lebanonensis*	Phote-HrTH	pELTFSPDWa	975.4571	XP_030380311.1
Brachycera, Cyclorrapha, Schizophora, Calyptrate	Glossinidae	*Glossina morsitans*	Phote-HrTH Glomo-AKH	pELTFSPDWa pELTFSPGWa	975.4571 917.4516	This study; (25, 36, 37); AEH25942.1 & AEH25941.1
		*Glossina fuscipes*	Phote-HrTH	pELTFSPDWa	975.4571	This study
			Glomo-AKH	pELTFSPGWa	917.4516	
		*Glossina austeni*	Phote-HrTH	pELTFSPDWa	975.4571	This study
			Glomo-AKH	pELTFSPGWa	917.4516	
		Other *Glossina* species^c^	Phote-HrTH	pELTFSPDWa	975.4571	(38)
			Glomo-AKH	pELTFSPGWa	917.4516	
Brachycera, Cyclorrapha, Schizophora, Calyptrate	Muscidae	*Musca domestica*	Phote-HrTH	pELTFSPDWa	975.4571	This study; XP_005178897.1
		*Musca autumnalis*	Phote-HrTH	pELTFSPDWa	975.4571	This study
		*Stomoxys calcitrans*	Phote-HrTH	pELTFSPDWa	975.4571	A0A1I8QC11
		*Haematobia irritans*	Phote-HrTH	pELTFSPDWa	975.4571	PGFW01000414.1
Brachycera, Cyclorrapha, Schizophora, Calyptrate	Anthomyiidae	*Fucellia capensis* *Delia radicum*	Phote-HrTH Phote-HrTH	pELTFSPDWa pELTFSPDWa	975.4571 975.4571	This study (39, 40); B3EWM7.1
Brachycera, Cyclorrapha, Schizophora, Calyptrate, Oestroidea	Sarcophagidae	*Sarcophaga (carnaria*?)	Phote-HrTH	pELTFSPDWa	975.4571	This study
		*Sarcophaga (= Neobellieria) bullata*	Phote-HrTH	pELTFSPDWa	975.4571	(30, 41, 42); TMW51522.1
		*Sarcophaga crassipalpis*	Phote-HrTH	pELTFSPDWa	975.4571	(17)
Brachycera, Cyclorrapha, Schizophora, Calyptrate, Oestroidea	Rhinophoridae	*Paykullia maculate*	Phote-HrTH	pELTFSPDWa	975.4571	NDXZ01142068.1
Brachycera, Cyclorrapha, Schizophora, Calyptrate, Oestroidea	Calliphoridae	*Calliphora vicina*	Phote-HrTH	pELTFSPDWa	975.4571	This study; JXOT01195109.1
		*Cochliomyia hominivorax*	Phote-HrTH	pELTFSPDWa	975.4571	PYHX01001830.1
		*Lucilia cuprina*	Phote-HrTH	pELTFSPDWa	975.4571	This study; (42) XP_023304778.1
		*Lucilia sericata*	Phote-HrTH	pELTFSPDWa	975.4571	JXPF01042013.1
		*Protophormia (= Phormia) terraenovae*	Phote-HrTH	pELTFSPDWa	975.4571	(21, 43)
		*Phormia regina*	Phote-HrTH	pELTFSPDWa	975.4571	(44); MINK01172052.1

* *Mature peptide sequence: in the case of sequences derived from nucleotide databases, the post-translational modifications (i.e., blocked termini) are deduced from the characteristic features of previously-sequenced AKHs. Note that the N-terminal pyroglutamic acid (pE) can arise from the cyclization of a glutamine (Q) or, more rarely, from a glutamate (E) residue. In the case of prepro-AKH sequences translated from mRNA, all have a glutamine amino acid residue at position 1 of the uncleaved AKH sequence. Novel peptide sequences deduced from data mining are consecutively numbered (1–4)—note that we have not confirmed the data experimentally*.

** *Published work or database accession number*.

a *Other Anopheles species (accession no.): A. coluzzii (A0A182LR92); A. stephensi (A0A182Y983); A. albimanus (A0A182FZY6); A. arabiensis (A0A182IIN3); A. atroparvus (A0A182J042); A. christyi (A0A182K374); A. culicifacies (A0A182MX76); A. darlingi (A0A158N7M0-1); A. epiroticus (A0A182PMF71); A. funestus (A0A182S506); A. melas (A0A182TPX4); A. merus (A0A182VPI3); A. minumus (A0A182WR25); A. quadriannulatus (A0A182XUQ9); A. sinensis (A0A084VYF4-1)*.

b *Other Drosophila species (accession no.): D elegans (XP_017127434.1); D. virilis (XP_002046309.1); D. novamexicana (XP_030561075.1); D. serrata (Sequence ID: XP_020807712.1); D. pseudoobscura pseudoobscura (XP_001353031.1); D. guanche (SPP77431.1); D. obscura (XP_022220454.1); D. kikkawai (XP_017019402.1); D. willistoni (XP_002068218.1); D. ananassae (XP_001957722.1); D. arizonae (XP_017860946.1); D. busckii (XP_017842867.1); D. mojavensis (XP_002012181.1); D. hydei (XP_023165811.1); D. bipectinata (XP_017101344.1); D. takahashii (XP_017009635.1); D. rhopaloa (XP_016978805.1); D. suzukii (XP_016943637.1); D. yakuba (XP_002093648.1); D. eugracilis (XP_017071536.1); D. biarmipes (XP_016966107.1); D. sechellia (XP_002035290.1); D. erecta (XP_001971844.1); D. simulans (XP_002083583.1)*.

c *Other Glossina species (gene identification number): G. pallidipes (GPAI036121; GPAI049064); G. austeni (GAUT013261; GAUT013267); G. palpalis (GPPI030617; GPPI030614); G. fuscipes (GFUI054167; GFUI054166); G. brevipalpis (GBRI027509; GBRI045557)*.

*** *A tipulid species that is not T. paludosa, and most likely T. oleracea (although not confirmed by a dedicated taxonomist) was collected in Crete, and the AKH complement was elucidated via MS*.

*The phylogenetic outline is as per Wiegmann et al. (8)*.

In the malaria mosquito, *Anopheles gambiae* (Family: Culicidae), genomic information, gene cloning and physiological experimentation led to the identification of a hypertrehalosemic hormone as an octapeptide with the sequence pELFTPAW amide [= Anoga-HrTH, (27, 46, 47)]. Similar genomic and immunocytochemical methods identified an AKH with the sequence pELTFTPSW amide (= Aedae-AKH) in the CC of the yellow fever mosquito, *Aedes aegypti* (Family: Culicidae), confirmed by mass spectrometric measurements (25, 26, 48). An AKH gene cloned from the West Nile virus vector, *Culex pipiens* = *C. quinquefasciatus* yielded an identical AKH, i.e., Aedae-AKH (25). Interestingly, Aedae-AKH as mature peptide was detected and sequenced by mass spectrometry in the alder fly *Sialis lutaria* which belongs not to Diptera but to the order Megaloptera (49). Kaufmann et al. (25) identified two AKH genes when mining publicly accessible genetic data from the tsetse fly *Glossina morsitans* (Diptera, Family: Glossinidae), the deduced mature peptides is the well-known Phote-HrTH and a novel octapeptide with the sequence pELTFSPGW amide (= Glomo-AKH); both of these genes were later cloned (36), and annotated during whole genome sequencing (50) of *G*. *morsitans*, while the mature peptide sequences were verified by mass spectrometry (37). Functionally, Phote-HrTH increased the amount of lipids released from fat body tissue of adult female *G*. *morsitans in vitro* (36); the function of Glomo-AKH was not physiologically investigated.

From the above, it is evident that there is some sort of group-specificity in the AKH family peptide sequences of the Diptera investigated to date, but there is also variation in the number of sequences per species and even in the length of the peptide sequence. Further elaboration of AKH sequences may also be useful in ascertaining the overlap of sequence identity in beneficial and pest dipteran species for the potential of finding a suitable lead for developing peptide mimetics that would target specifically pest dipterans. An additional aim of the current study therefore is to provide a comprehensive list of putative AKH sequences from Diptera by mining publicly-accessible data bases.

## Materials and Methods

### Insects

Adult specimens of unknown age and both sexes were used in this study. Fly species were either caught in the field by netting, were purchased from breeders or were received as a gift from a research institution. In total 19 species of Diptera were studied. Details of the fly species and the taxonomic affiliations are given below. For the latter, the phylogenetic outline given by Pape et al. (51) and Wiegmann et al. (8) were followed (see also section Introduction).

#### Suborder Lower Diptera (Formerly: Nematocera), Infraorder Tipulomorpha

Two species of crane fly (Family: Tipulidae) were investigated. Specimens of *Tipula paludosa* were caught on a meadow in Groß Raden in Mecklenburg-Western Pomerania (Mecklenburg-Vorpommern), Germany, by netting. Specimens of the other species (very likely *Tipula oleracea* based on morphological characters that we could ascertain) were caught on the wall of a hotel complex in northern Crete close to Heraklion.

#### Suborder Brachycera, Infraorder Stratiomyomorpha

One species of soldier fly (Family: Stratiomyidae) was investigated. Specimens of *Hermetia illucens* were purchased from Illucens GmbH, Ahaus, Germany.

#### Suborder Brachycera, Superfamily Syrphoidea

Six species of hover fly (Family: Syrphidae) were investigated. Specimens of *Volucella pellucens, Volucella zonaria, Chrysotoxum cautum*, and *Eristalis* ssp. were collected by netting from flowers of wild oregano (*Origanum vulgare*) in July and August 2017 and 2019 around Bad Iburg and Hagen, Germany. Specimens of *Eristalis tenax* and *Allograpta fuscotibialis* were netted on flowers in a private garden in Cape Town, South Africa.

#### Suborder Brachycera, Superfamily Tephritoidea

One species of fruit fly (Family: Tephtritidae) was investigated. Specimens of the Mediterranean fruit fly (“medfly”) *Ceratitis capitata* were a gift from the Fruitfly Africa Medfly rearing facility in Stellenbosch, South Africa.

#### Suborder Brachycera, Superfamily Hippoboscoidea

Three species of tsetse fly (Family: Glossinidae) were investigated. Specimens of *Glossina fuscipes* and *G. austeni* came from the Veterinary Institute in Onderstepoort, South Africa, whereas *G. morsitans morsitans* were a gift from the International Atomic Energy Agency in Vienna, Austria.

#### Suborder Brachycera, Superfamily Muscoidea

Two species of the house fly (Family: Muscidae) and one species of kelp fly (Family: Anthomyiidae) were investigated. Specimens of *Musca domestica* were a gift from Clintech (Pty), Ltd., Bryanston, South Africa; *Musca autumnalis* was caught in the wild in August 2019 around Bad Iburg, Germany, and the kelp fly *Fucellia capensis* was caught by netting on rotting kelp on the beach of Muizenberg, South Africa, in August 2019.

#### Suborder Brachycera, Superfamily Oestroidea

Two species of blowfly (Family: Calliphoridae) and one of flesh fly (Family: Sarcophagidae) were investigated. Specimens of *Calliphora vicina* and *Sarcophaga* ssp. (very likely *carnaria*) were collected during July and August 2017 around Osnabrück, Germany, and *Lucilia cuprina* was a gift of Clintech (Pty), Ltd., Bryanston, South Africa.

#### Species of the Order Mecoptera

Adults of both sexes of the common scorpion fly, *Panorpa communis*, were collected by netting during July 2017 around Bad Iburg, Germany.

### Biological Assays

Conspecific metabolic bioassays were performed with adult specimens of both sexes of the soldier fly *Hermetia illucens*, of which a larger number of individuals was available. Flies were acclimatized for about 1 h before experimentation at ambient temperature (22 ± 1°C), each in a 15 ml plastic container with water-soaked cotton wool pieces. After this resting period, 0.5 μl of hemolymph was sampled dorsally from the thorax with a disposable glass microcapillary (Hirschmann Laborgeräte, Eberstadt, Germany), and the hemolymph was blown into concentrated sulfuric acid to measure vanillin-positive material (= total lipids) or anthrone-positive material (= total carbohydrates) according to the phosphovanillin method (52) and anthrone method (53), respectively, as modified by Holwerda et al. (54). Subsequently, the flies were injected ventrolaterally into the abdomen with 3 μl of either water (a control for assessing stress effects of handling), a crude corpora cardiaca extract, or a synthetic peptide using a Hamilton fine-bore 10 μl syringe. A second hemolymph sample was taken 90 min post-injection from the same individuals under resting conditions.

### Dissection of Corpora Cardiaca, Peptide Extraction, Mass Spectrometry, and Sequence Analysis

Corpora cardiaca (CCs) were microdissected from the head/neck region of adult flies from each species under investigation using a stereomicroscope at about 20- to 30-fold magnification; CCs were placed into 80% v/v methanol. Peptide material were extracted from the dissected CCs as outlined previously (55). The vacuum-centrifuged dried extracts were dissolved in 50 μl of aqueous formic acid for liquid chromatography tandem positive ion electrospray mass spectrometry (LC-MS^n^) on an LTQ XL linear ion trap instrument (Thermo Fisher Scientific, San Jose, CA), as described in detail previously (56).

For conspecific biological assays with the black soldier fly (see section Biological Assays), the dried CCs extracted from *H. illucens* were reconstituted in distilled water for injection into the flies.

### Synthetic Peptides

The following peptides were previously synthesized by Peninsula Laboratories (Belmont, CA, USA), Pepmic Co. Ltd. (Suzhou, China), or custom-synthesized by Dr. Kevin Clark (University of Georgia, Athens, USA): Phote-HrTH (= Drome-HrTH; pELTFSPDW amide), Glomo-AKH (pELTFSPGW amide), Tabat-AKH (pELTFTPGW amide), Tabat-HoTH (pELTFTPGWGY amide), Anoga-HrTH (pELTFTPAW amide), and Aedae-AKH (pELTFTPSW amide). The novel peptides of this study, Eriss-CC (pELTFSAGW amide), Volpe-CC (pELTFSPYW amide), Tippa-CC-I (pELTYSPSW amide) and Tippa-CC-II (pELTFSPSW amide) were all custom-synthesized by Pepmic Co. Ltd.

### Mining of AKH Sequences From Publicly Available Data Bases

The primary sequence of AKH family peptides in dipteran species were investigated by mass spectrometry (see Section Dissection of Corpora Cardiaca, Peptide Extraction, Mass Spectrometry, and Sequence Analysis). To compare and analyse Dipteran AKHs in a phylogenetic manner, we identified further AKH sequences from other dipteran species via literature searches (i.e., from previously published texts), as well as via bioinformatics. Such, *in silico* searches of Dipteran protein, genomic and/or EST databases were conducted to identify translated amino acid sequences and transcripts encoding putative AKH peptide precursors.

Some AKH sequences were retrieved from VectorBase (https://www.vectorbase.org/), which is a Bioinformatics Resource for invertebrate vectors of human pathogens. The searches for AKH genes were conducted using the “search term” function, or via the BLAST (Basic Local Alignment Search Tool) search function whereby the nucleotide sequence of the *Glossina morsitans* AKH was inserted. From the search results, the AKH peptide sequence contained within the deduced prepro-hormones were predicted from homology to known insect AKH isoforms. The associated UniProt accession numbers (https://www.uniprot.org/uniprot) were recorded. The UniProt Knowledgebase (UniProtKB) is the publicly available central hub for the collection of functional information on proteins.

The remainder of putative AKH sequences were obtained via homology searches using BLAST from the National Center for Biotechnology Information site (https://blast.ncbi.nlm.nih.gov/); AKH peptide precursors from *G. morsitans* were used as BLAST query. For all searches resulting in sequence identifications, the BLAST score and BLAST-generated E-value for significant alignment were considered.

## Results and Discussion

### Function of Dipteran AKH: The Black Soldier Fly as a Test Case

We conducted a conspecific biological assay with only one dipteran species to show proof of principle that the CC extracts of dipteran species do have hypertrehalosemic biological activity. Unambiguous results reveal that conspecific CC extracts of the black soldier fly *H. illucens* injected into resting adults had a small but statistically significant hypertrehalosemic effect (Table 2). Such a hypertrehalosemic effect was also shown when the endogenous AKH octapeptide of the black soldier fly, Tabat-AKH, was injected, whereas the lipid concentration was not affected (Table 2). These results confirm previous data on the action of AKH peptides in a blowfly (22) and the malaria mosquito (47), viz. the mobilization of carbohydrates. When working with a crude CC extract, one could argue that the extract contains, of course, all other putative neuropeptide hormones. Although we are not aware of any neuropeptide that is in higher concentration in the CC and has a clear effect of increasing carbohydrates or lipids except the AKHs, we can only assume that the measured effect was induced by an AKH.

**Table 2:** Biological activity of a crude methanolic extract of corpora cardiaca from the black soldier fly (Hermetia *illucens*), and the synthetic peptide Tabat-AKH in homologous bioassays.

**Treatment**	**Hemolymph carbohydrates (mg ml**^****−1****^**)^a^**					**Hemolymph lipids (mg ml**^****−1****^**)^a^**				
	***n***	**0 min**	**90 min**	**Difference**	**p**^**b**^	***n***	**0 min**	**90 min**	**Difference**	**p**^**b**^
Control (3 μI distilled water)	18	12.9 ± 4.7	13.0 ± 4.9	0.2 ± 1.7	NS	11	14.3 ± 6.7	13.4 ± 7.4	−0.9 ± 2.2	NS
*H. illucens* CC (0.5 gland pair equiv.)	6	11.5 ± 3.5	15.7 ± 5.0	4.2 ± 2.0	0.002			Not determined		
Tabat-AKH (10 pmol)	23	13.1 ± 4.6	17.4 ± 6.5	4.3 ± 3.8	0.00001	11	14.0 ± 7.7	13.6 ± 7.5	−0.5 ± 1.6	NS

a *Data are presented as Mean ± SD*.

b *Paired t-test was used to calculate the significance between pre- and post-injection. NS, not significant*.

However, it is clear from studies on other Diptera that these peptides can also mobilize lipids in certain fly species (20, 36). Thus, as reported for other insect orders, it is the metabolic machinery of the species under investigation and the metabolic needs of an insect that determines activation of a lipase and/or a phosphorylase by AKHs (18, 57).

### Mass Spectral Analyses of AKHs Reveal Octapeptides in Major Clades of Diptera

Methanolic CC extracts from all 19 fly species were analyzed by mass spectrometry. Here we will show a few exemplary cases; we chose species from the infraorders Tipulomorpha and Stratiomyomorpha, as well as from the superfamilies Syrphoidea and Tephritoidea.

#### Tipulomorpha *Tipula paludosa*

**Figure 1:**
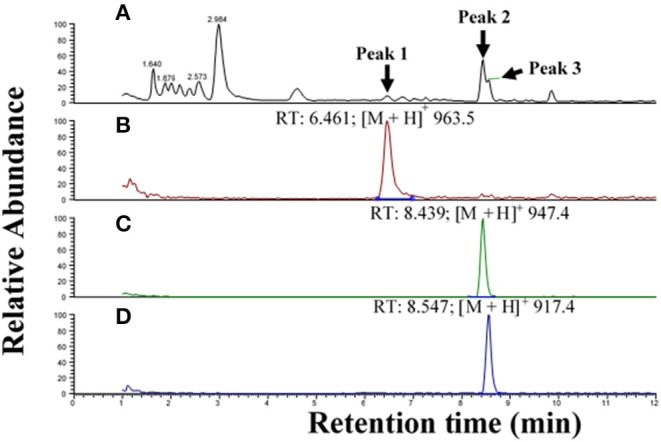
Liquid chromatographic (LC) positive electrospray ionization (+ESI) mass spectrometric (MS) analysis of an extract from corpus cardiacum material of the crane fly *Tipula paludosa*. **(A)** Base peak chromatogram obtained by LC-MS analysis showing detection of three AKH peptides labeled 1, 2, and 3 at 6.46, 8.44, and 8.55 min, respectively. **(B)** The extracted LC-MS chromatogram of peak 1 in **(A)** with [M + H]^+^ at *m/z* 963.5. **(C)** The extracted LC-MS chromatogram of peak 2 in **(A)** with [M + H]^+^ at *m/z* 947.4. **(D)** The extracted LC-MS chromatogram of peak 3 in **(A)** with [M + H]^+^ at *m/z* 917.4.

**Figure 2:**
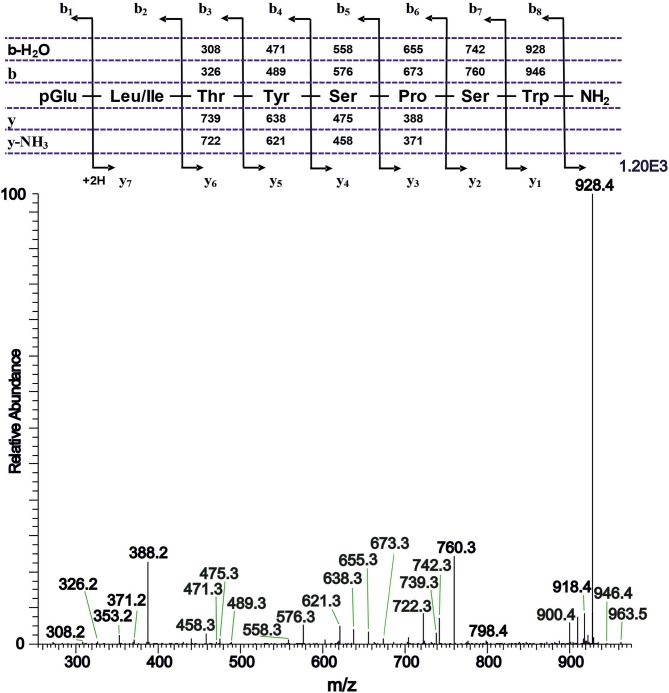
A collision-induced dissociation (CID) tandem MS + ESI spectrum of the ion [M + H]^+^ = 963.5 in Figure 1B from the CC of *T. paludosa*. The inset shows the proposed peptide sequence together with the b, y, b-H_2_O and y-NH_3_ diagnostic fragment ions observed in the MS^2^ spectrum. This is a novel AKH.

**Figure 3:**
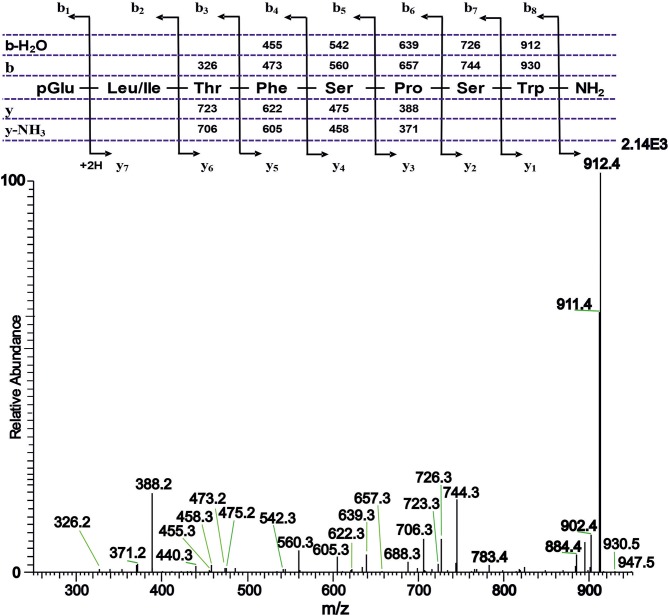
A collision-induced dissociation (CID) tandem MS + ESI spectrum of the ion [M + H]^+^ = 947.4 in Figure 1C from the CC of *T. paludosa*. The inset shows the proposed peptide sequence together with the b, y, b-H_2_O and y-NH_3_ diagnostic fragment ions observed in the MS^2^ spectrum. This is a novel AKH.

**Figure 4:**
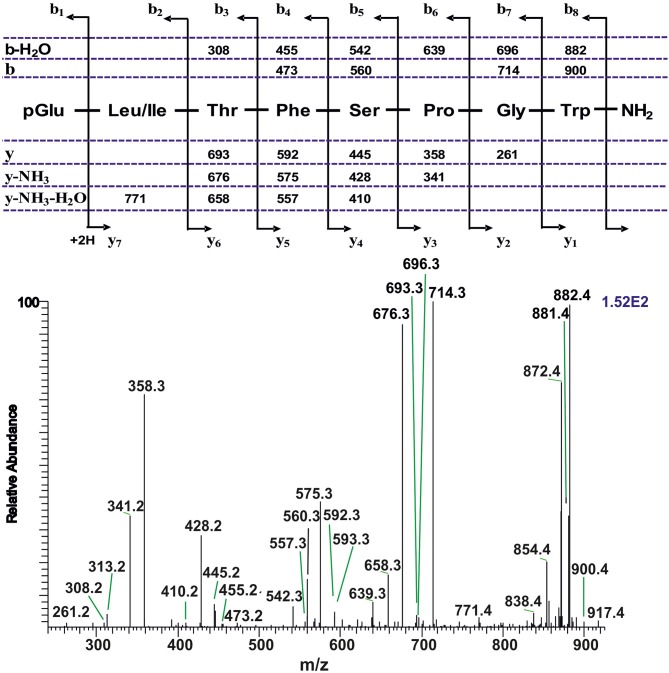
A collision-induced dissociation (CID) tandem MS + ESI spectrum of the ion [M + H]^+^ = 917.4 in Figure 1D from the CC of *T. paludosa*. The inset shows the proposed peptide sequence together with the b-, and y-type diagnostic fragment ions observed in the MS^2^ spectrum.

The CC extract of the crane fly *T. paludosa* was fractionated (separated) by reversed-phase liquid chromatography (LC) and the peptides detected by positive ion electrospray mass spectrometry (MS). Figure 1A shows the base peak chromatogram; Figures 1B–D depict extracted mass peaks of AKHs at 6.46, 8.44, and 8.55 min, respectively, with the corresponding [M + H]^+^ mass ions at *m/z* 963.5, 947.4, and 917.4, respectively. The primary structures of these peak materials were deduced from the tandem MS^2^ spectra obtained by collision-induced dissociation (CID) of the respective *m/z* ions. The spectrum of *m/z* 963.4 (Figure 2) with clearly defined b, y, b-H_2_O, y-NH_3_, and other product ions allowed an almost complete assignment of a typical octapeptide member of the AKH family under the assumption that the peptide has a characteristic pyroglutamate residue at the N-terminus (see schematic inset in Figure 2). All other amino acids are assigned except at position two where the remaining mass of 113 can be accredited to one of the isomers leucine or isoleucine. Such a peptide with the sequence pGlu-Ile/Leu-Thr-Tyr-Ser-Pro-Ser-Trp amide had never been found in any insect, to date. It was hence code-named Tippa-CC-I (*Tipula paludosa* CC peptide I) and awaited clarification of the amino acid residue in position 2 through co-elution experiments with a synthetically made Leu^2^ analog of the peptide (see below). The spectrum of *m/z* 947.4 (Figure 3) led to the interpretation of another octapeptide with the sequence pGlu-Ile/Leu-Thr-Phe-Ser-Pro-Ser-Trp amide which is also novel and, thus, called Tippa-CC-II with the same ambiguity about position 2 in the primary sequence. The spectrum of the third peptide at *m/z* 917.4 (Figure 4) resulted in the sequence interpretation pGlu-Ile/Leu-Thr-Phe-Ser-Pro-Gly-Trp amide which, with Leu^2^, is well-known under the name Glomo-AKH as one of the two peptides found in the tsetse fly (see section Introduction; Table 1). All three peptides were synthesized as Leu^2^ isomer and co-elution experiments were performed; previously we had established that the isobaric Leu/Ile peptides have different LC retention times (58, 59). As depicted in Figures S1A–I all three synthetic peptides had identical retention times to the natural peptides in the CC extract and have, therefore, the correctly assigned primary peptide sequence, confirming that leucine is the second amino acid residue also of the novel Tippa-CC-I and II. The same three masses were also identified from the tipulid species caught on Crete and support the finding of these 3 octapeptides in this infraorder.

When we look at the primary structure of the three AKHs of *T. paludosa* and the other tipula species, it is obvious that they are closely related to each other and to the AKH known as Phote-HrTH from *Phormia* and *Drosophila* (Table 1).

#### Stratiomomorpha *Hermetia illucens*

An extract from the CC of the black soldier fly shows a peak with a retention time of 8.88 min (Figure S2A) which corresponds in MS analysis to an [M + H]^+^ ion of *m/z* 931.4 (Figure S2B). The CID spectrum gave clear product ions and led to the interpretation of an AKH with the primary structure pGlu-Ile/Leu-Thr-Phe-Thr-Pro-Gly-Trp-amide (Figure S2C) of which the Leu^2^ form was established by a co-elution experiment (Figures S2D–F). This peptide is known as Tabat-AKH and found in *Tabanus atratus* (19). Surprisingly, genetic work has proposed another peptide for this species, i.e., pGlu-Leu-Thr-Phe-Thr-Gly-Gln-Trp-amide, which differs at position 6 (Gly instead of Pro) and 7 (Gln instead of Gly), and in its calculated protonated mass of 962.4730 [see Table 1; (28)]. Although there were many other prominent peak material eluting from the CC extract in the current study (Figure S2A), these peaks did not correspond with the calculated mass of 962.47, nor did they relate to an AKH peptide sequence. Since there has never been a glycine residue found at position 6 in any of the 90 AKHs known so far (G. Gäde personal communication), we suggest that there may be a misread in the genetic code and, hence, do not currently accept this sequence as a novel one until it has been confirmed (or refuted) by mass spectrometric methods.

#### Syrphoidea: *Eristalis* ssp

**Figure 5:**
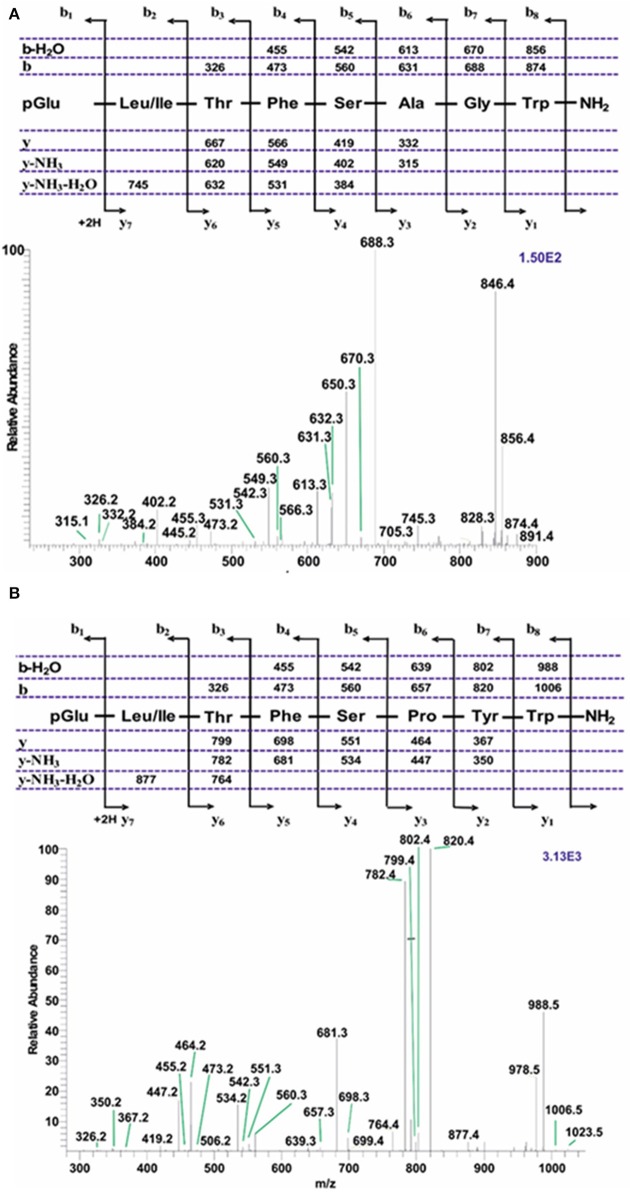
Liquid chromatographic (LC) positive electrospray ionization (+ESI) mass spectrometric (MS) analysis of an extract from corpus cardiacum material of a mixture of hoverfly species from the genus *Eristalis*. **(A)** A collision-induced dissociation (CID) tandem MS + ESI spectrum of the ion [M + H]^+^ = 891.4. The inset shows the proposed peptide sequence together with the b- and y-type diagnostic fragment ions observed in the MS^2^ spectrum. This is a novel AKH. **(B)** A collision-induced dissociation (CID) tandem MS + ESI spectrum of the ion [M + H]^+^ = 1023.5. The inset shows the proposed peptide sequence together with the b- and y-type diagnostic fragment ions observed in the MS^2^ spectrum. This is a novel AKH.

An extract from the CC of a number of *Eristalis* specimens, possibly a mixture of a few species that are difficult to distinguish taxonomically, gave information of four clearly identified AKHs. The extracted chromatograms reveal three AKHs with near-identical retention times but with different [M + H]^+^ ions of *m/z* 891.4, 917.4, and 975.5, the fourth AKH with [M + H]^+^ ion of *m/z* 1023.5 had a longer retention time (Figures S3A–E). The CID spectra gave clear evidence for the sequence of the respective AKHs. Here we show only the CIDs for [M + H]^+^ 891.4 and 1023.5 because they represent novel AKHs. The peptide with [M + H]^+^ 917.4 (= Glomo-AKH) has been dealt with in the earlier example (see Tipulomorpha: *Tipula paludosa*) while [M + H]^+^ 975.5 (= Phote-HrTH) will be discussed later (see Tephritoidea: *Ceratitis capitata*). The primary structure of an AKH representing [M + H]^+^ 891.4 was deduced as pGlu-Leu/Ile-Thr-Phe-Ser-Ala-Gly-Trp amide (Figure 5A) and the one with [M + H]^+^ 1023.5 was assigned the sequence pGlu-Leu/Ile-The-Phe-Ser-Pro-Tyr-Trp amide (Figure 5B). The ambiguity at position two was solved to the presence of Leu in both cases by co-elution experiments with the synthetic peptide (Figures S3F–Q). Both peptides are novel, thus have not been found in any insect before and we assign them the code-name Eriss-CC for the peptide with MH^+^ 891.4 because it occurs in an *Eristalis* subspecies (see Table 1: *Eristalis* subspecies 2), and Volpe-CC for the peptide with MH^+^ 1023.5 as this peptide was discovered in the unambiguously defined species *Volucella pellucens* and all other syrphid species in the current study (Table 1).

#### Tephritoidea *Ceratitis capitata*

An extract from the CC of the fruit fly shows only one peak with a retention time of 8.81 min (Figure S4A) which corresponds in MS analysis to an [M + H]^+^ ion of *m/z* 975.4 (Figure S4B); the CID (Figure S4C) determines a sequence of pGlu-Leu/Ile-Thr-Phe-Ser-Pro-Asp-Trp amide. Co-elution with synthetic peptide (Figures S4D–F) unequivocally determines Leu at position 2 and, hence, characterizes this peptide as Phote-HrTH found previously in certain flies [(21, 23); see Table 1].

#### AKHs of Diptera Are Order-Specific

The CC extracts of all the other Diptera investigated in the current study were analyzed in the same way as in the above examples. The results are given in Table 1. In this table we present the taxonomic units of Diptera in the phylogenetic relationship as outlined by Wiegmann et al. (8). Additionally, previously published data on AKH sequences are incorporated in this table as are sequences that were “mined” from genomic or transcriptomic data sets as outlined in Materials and Methods.

It is apparent from the combined data sets in Table 1 that there are a few main points to be made about the AKHs in Diptera which will be discussed in more detail hereafter: (i) Dipteran AKHs are octapeptides. There is only one decapeptide exception. (ii) Current mass spectral investigations find 4 novel octapeptides from fly corpora cardiaca; an additional 4 novel octapeptides are found through data mining. (iii) Dipteran AKHs are specific for the order. There are only two exceptions.

To date, we know roughly 90 sequences of AKHs from insects. There is a clear bias toward the production of octapeptides as only one third of the known AKHs are decapeptides and only three are nonapeptides. Most of the decapeptide AKHs are found in the orders Hymenoptera, Hemiptera and Caelifera, while the nonapeptides are in Lepidoptera and Hemiptera. The order under scrutiny here, the Diptera, contain up to now only a single AKH decapeptide, viz. Tabat-HoTH in the horse flies. The presence of Tabat-HoTH can be well-explained by gene duplication since this decapeptide has the same amino acid sequence as the accompanying octapeptide in the horse fly but is elongated by two amino acids at the C- terminus. It remains to be seen whether another genus of the tabanids also has such a complement of AKHs. Unfortunately, our efforts with an extract prepared from long-frozen CCs of the common horse fly, *Haematopota fluvialis*, did not yield any mass spectral data for inclusion in the current work. Although gene duplication has taken place in other dipteran species, such as in crane flies and hover flies as shown in the current work, this has not led to decapeptides but mutations to other octapeptides.

Before we started this study, 6 AKHs were known from the order Diptera (see history in section **Introduction**). The current study adds 8 further sequences: 4 novel AKH peptides (2 in the tipulids and 2 in the syrphids) found by unequivocal mass spectrometric studies, and 4 novel sequences (distributed in Culicomorpha, Stratiomyomorpha, Asiloidea, Diopsidae, and Drosophilidae) from data mining of public data bases—although it should be cautioned that the “mined” sequences have not been substantiated by mass spectrometry or peptide chemistry. Three of the four characterized novel AKHs and 1 of the mined AKHs share the same N-terminal pentapeptide pELTFS, while the fourth novel AKH share pELTYS with one of the mined AKHs (Table 1). The remaining 2 of the 4 novel peptides found by data mining have the pentapeptide sequence pELTFT (Table 1). A scan of the dipteran AKH sequences in Table 1 shows that the most prevalent amino acid residue in position 6 is proline, and there is a clear distribution pattern of AKHs with pELTFSP in lower diptera Tipulomorpha, while pELTFTP is present from the lower diptera Psychodomorpha until its last appearance in orthorraphan Brachycera Asiloidea, after which AKHs with pELTFSP reappear in Brachycera Syrphoidea onwards).

In contrast to almost all other insect orders it appears that AKHs in Diptera are relatively order-specific. Of the 14 AKHs now known from Diptera, only two are found outside of this order, viz. Aedae-AKH which is found in the only Megaloptera species investigated to date (49), and a new discovery from the current work: Glomo-AKH is found in a species from Mecoptera (see Putative Molecular Evolution of Dipteran AKHs). This is quite remarkable when considering the diversity and species-richness of Diptera. Such order-specificity is at the moment also known from Lepidoptera (60) but not from Coleoptera (59, 61) and Orthoptera (13, 62). We cannot, at this point, exclude the possibility that future investigations may reveal AKH structures that are common to Diptera and various other orders.

### Putative Molecular Evolution of Dipteran AKHs

**Figure 6:**
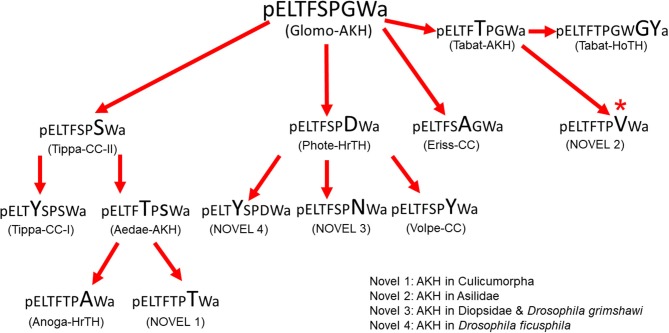
Hypothetical molecular evolution of adipokinetic peptides in Diptera. Glomo-AKH is assumed as ancestral peptide for this order. The amino acid substitution in each peptide is indicated in a larger font than in the peptide from which it is hypothetically derived. All substitutions are point mutations except the change from Tabat-AKH to the unconfirmed novel peptide 2 found by data mining. *The switch from Gly^7^ to Val^7^ requires two base changes.

If we want to speculate about the molecular evolution of dipteran AKHs we first have to find out what the putative ancestor AKH may be. For this it would be advantageous to know the AKHs of the closest relatives to the Diptera. According to Wiegmann et al. (63) the orders Mecoptera (scorpion flies) and Siphonaptera (fleas) are evolutionary closest and not the parasitic Strepsiptera as once postulated. Data mining revealed genomic information for a member of the Siphonaptera proposing a mature AKH of the cat flea, *Ctenocephalides felis*, with the sequence pELTFTPVW amide (XP_026477356.1), which is also a predicted AKH for the robber fly *Dasypogon diadema* (QYTT01077274.1; see Table 1). We did not find any data base information on Mecoptera and thus decided to include the common scorpion fly, *Panorpa communis*, into our study. The mass spectrometric data from the CC of this insect are clear: the AKH has the ionized mass (MH^+^) of 917.5 and the sequence of pELTFSPGW amide, thus Glomo-AKH (results not shown). Thus, both related orders have AKHs with sequences that also occur in Diptera, viz. Glomo-AKH in members of the Tipulidae, Syrphidae, and Glossinidae and the novel peptide (novel 2) in a member of the Asilidae (see Table 1). This may point to a common ancestor of the three orders with the sequence pELTFXPXW amide. Unfortunately, we do not have data on AKHs from the most basal fly families, that are species-poor and not very accessible, i.e., the Deuterophlebiidae and Nymphomyiidae (8). Currently, the AKHs known from Tipulidae are the ones from the most basal dipteran family. Since Glomo-AKH does also occur in the close relative order Mecoptera and in a number of dipteran families, we currently assume this molecule as parent to speculate and sketch a molecular evolution of the AKHs occurring in the various families of Diptera, as inferred by single point mutations. Figure 6 clearly shows that this hypothetical peptide evolution could have occurred without introducing any new sequences to the ones listed in Table 1. Additionally, only in one case indicated in the figure would two nucleotides of the triplet have to mutate. Although speculation, this scheme of putative evolution has all the known AKHs of the lower Diptera (Tipulomorpha and Culicomorpha) on one side (left) of the flow chart giving it some credibility.

As previously shown, e.g., for Odonata, sequences of mature AKHs do not allow an insight into deeper phylogenetic relationships but give some indications of relatedness (10, 64). For example, the three peptides of the Tipulidae are very similar, as are the four of the Syrphidae and also the three in Drosophilidae (see Table 1 and Figure 6). It is also clear that the vast majority of the cyclorraphan flies, which are monophyletic (8), only produce Phote-HrTH. It appears that quite a radiation of AKHs occurred early in the evolution of the Diptera, thus in the Tipulomorpha and Culicomorpha which are thought to have radiated in the Triassic (8). The next wave of radiation according to Wiegmann et al. (8) occurred in the early Jurrasic by the lower Brachycera including the clades Tabanomorpha, Stratiomyomorpha and Asiloidea all of which have their AKHs clustered together at the right side of Figure 6. The latest radiation involves the clade Schizophora between earliest Paleocene to Tertiary and includes the species in the Tephritoidea, Ephydroidea and Oestroidea which all produce Phote-HrTH and its closest relatives (see middle of Figure 6).

## Data Availability Statement

All datasets for this study are included in the article/Supplementary Material. Any other requests can be directed to Gerd Gäde, gerd.gade@uct.ac.za for metabolic data and Petr Šimek, simek@bclab.eu for mass spectrometric data.

## Author Contributions

GG: concept and design of the study, acquisition of insect species and synthetic peptides, interpretation of the data and writing the draft manuscript. HM: co-designed the study, data acquisition (biological assays, dissection of insect corpora cardiaca and preparation of extracts, mining data bases for AKH sequences), interpretation and analyses of data, writing and refining the draft manuscript. PS: mass spectrometric analyses, data interpretation, and drafting of MS Figures for the manuscript.

### Conflict of Interest

The authors declare that the research was conducted in the absence of any commercial or financial relationships that could be construed as a potential conflict of interest.
