# Supplementary material for: The Adipokinetic Peptides in Diptera: Structure, Function, and Evolutionary Trends
Source: Front Endocrinol (Lausanne). 2020 Mar 31;11:153. doi: 10.3389/fendo.2020.00153 (PMC7136388; doi:10.3389/fendo.2020.00153)
Supplement: Supplementary file 1 [file Data_Sheet_1.pdf]

SUPPLEMENTARY FIGURE S1. Confirmation of the AKH peptide structures in the crane fly *Tipula paludosa* corpus cardiacum extract by HPLC-MS co-elution of the three native peaks with the corresponding diluted synthetic AKH peptide. An extracted ion HPLC-MS chromatogram is depicted for each detected AKH.

Fig. S1 A - C. An LC-MS co-elution experiment of the CC extract-derived peptide 1 with MH^+ 963.4 from the crane fly spiked with the synthetic peptide: pELTYSPSW-NH_2; MH^+ = 963.4**.** The extracted chromatograms A-C reveal one prominent peak that co-incides with the retention time of the native peptide, thus indicating that the amino acid in position 2 is Leu, and not Ile. The identity of Tippa-CC-I is therefore pELTYSPSW-NH_2.

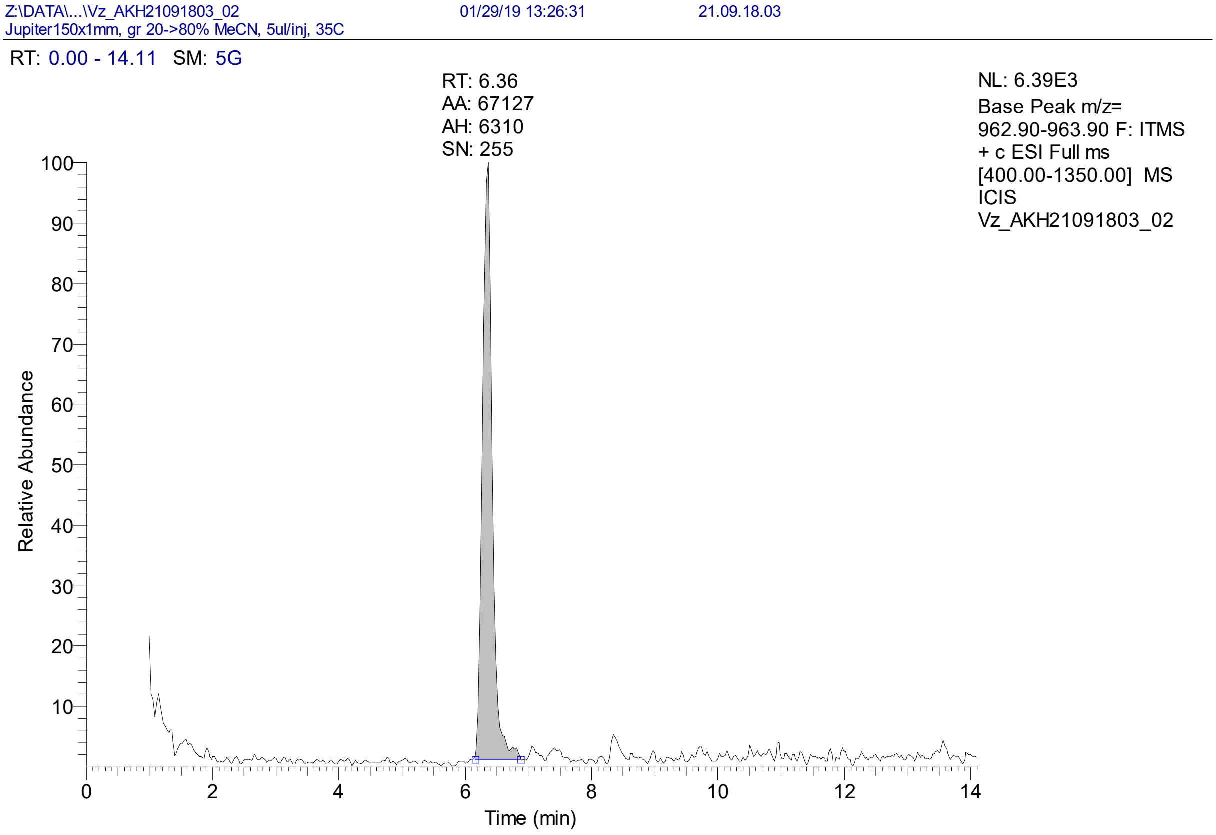

Fig. S1 A. LC-MS chromatogram of the synthetic novel AKH peptide pELTYSPSW-NH_2; MH^+ = 963.5**.**

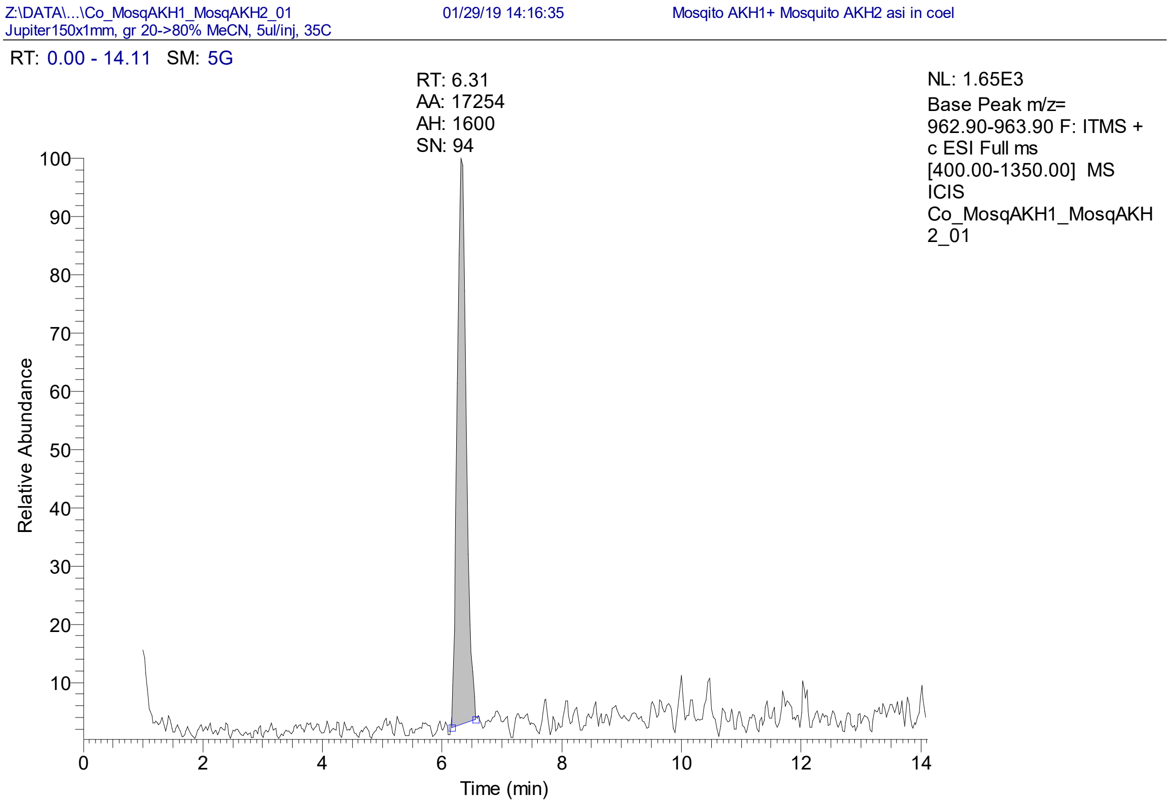

Fig. S1 B. LC-MS extracted chromatogram of the sample, *T. paludosa* corpus cardiacum Peak 1, with an extracted mass $MH^+ = 963.5$.

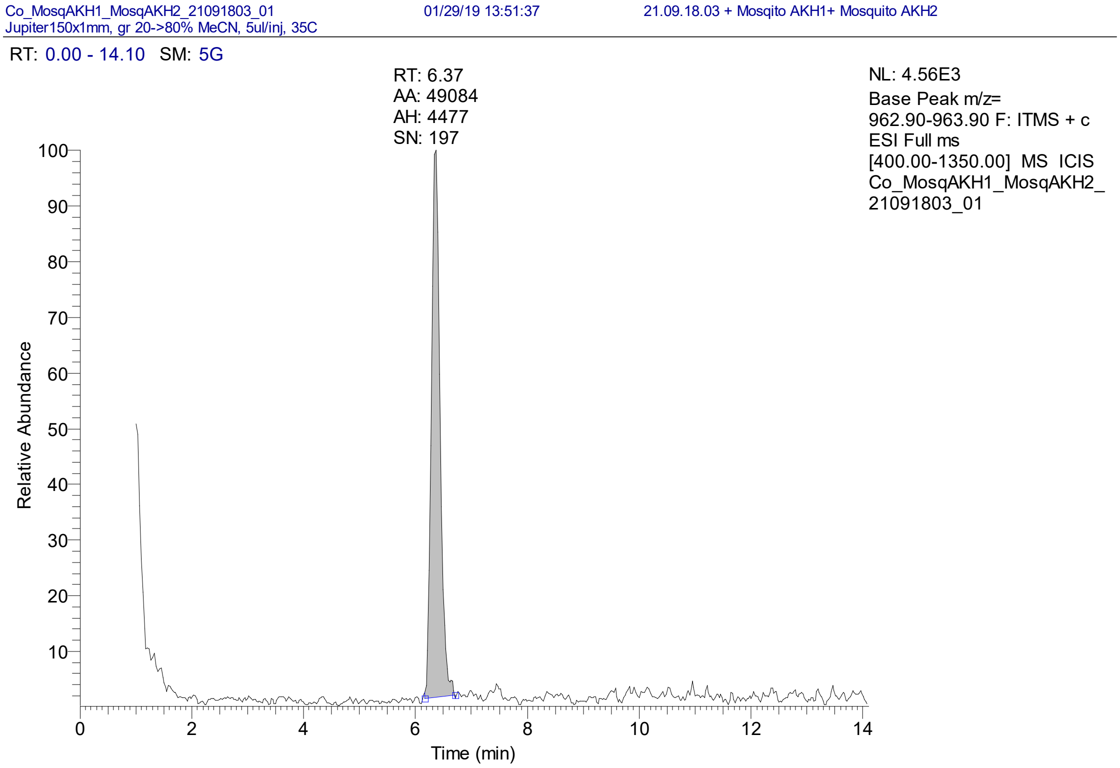

Fig. S1 C. LC-MS chromatogram of the sample, *T. paludosa* corpus cardiacum Peak 1 spiked with the synthetic novel AKH peptide pELTYSPSW-NH_2. The native Peak 1 coelutes with pELTYSPSW-NH_2 which is now code-named Tippa-CC-I.

Fig. S1 D - F. An LC-MS co-elution experiment of the CC extract-derived peptide 2 with MH^+ 947.4 from the crane fly spiked with the synthetic peptide: pELTFSPSW-NH_2; MH^+ = 947.4**.** The extracted chromatograms D-F reveal one prominent peak that co-incides with the retention time of the native peptide, thus indicating that the amino acid in position 2 is Leu, and not Ile. The identity of Tippa-CC-II is therefore pELTFSPSW-NH_2.

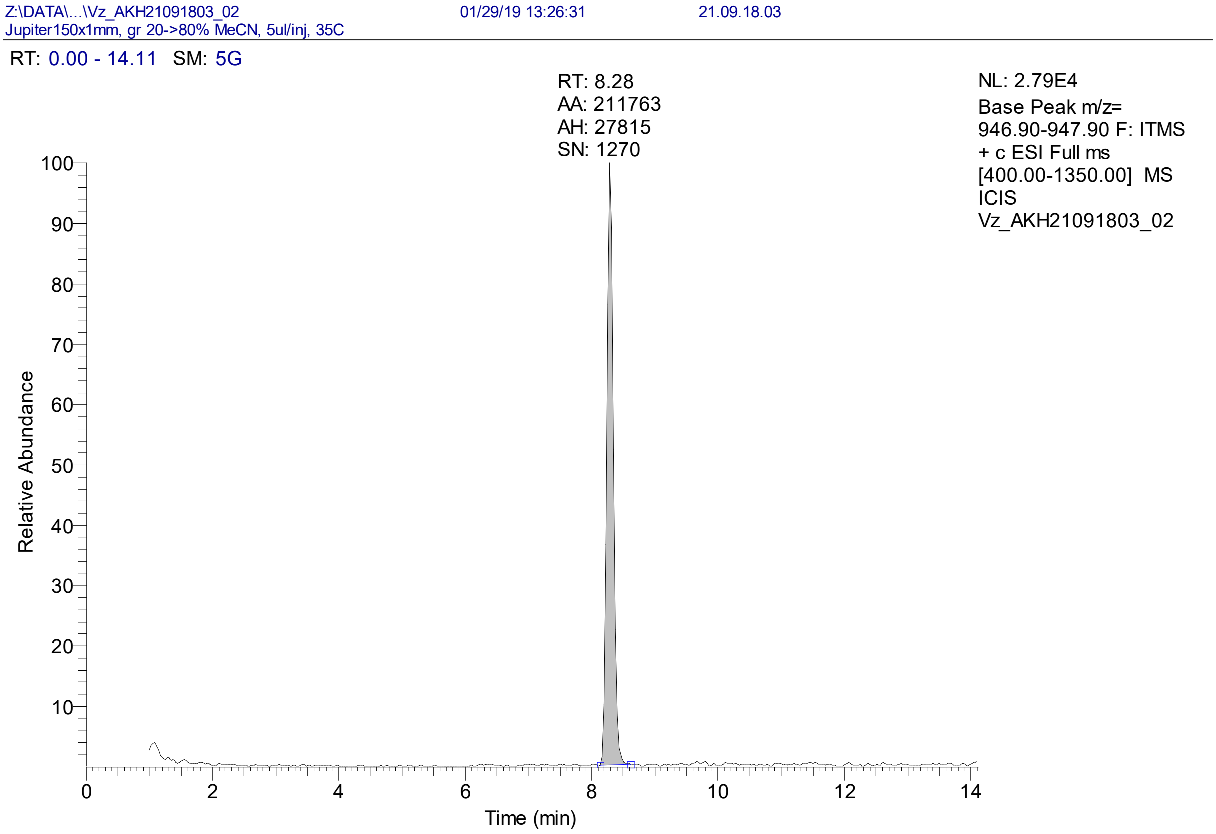

Fig. S1 D. LC-MS chromatogram of the synthetic novel AKH peptide pELTFSPSW-NH_2; MH^+ = 947.4

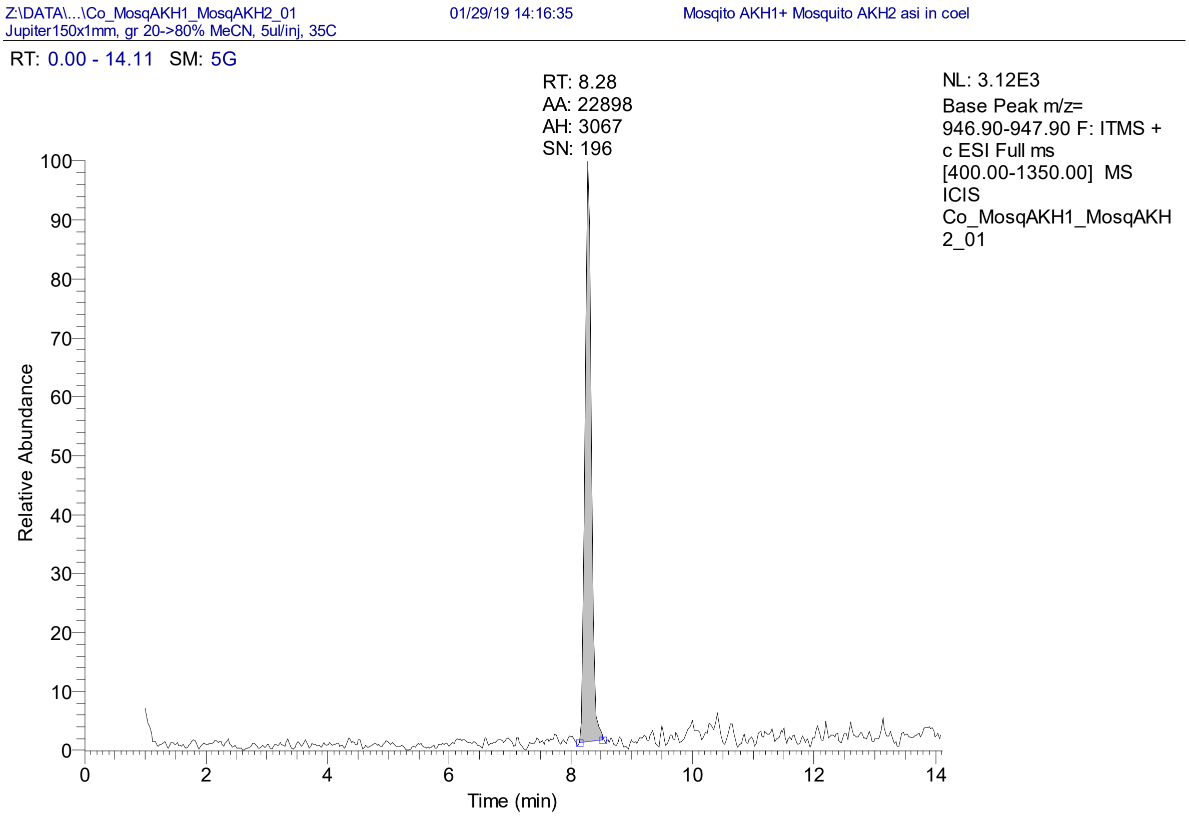

Fig. S1 E. LC-MS chromatogram of the sample, *T. paludosa* corpus cardiacum Peak 2, with an extracted mass MH^{+} = 947.4.

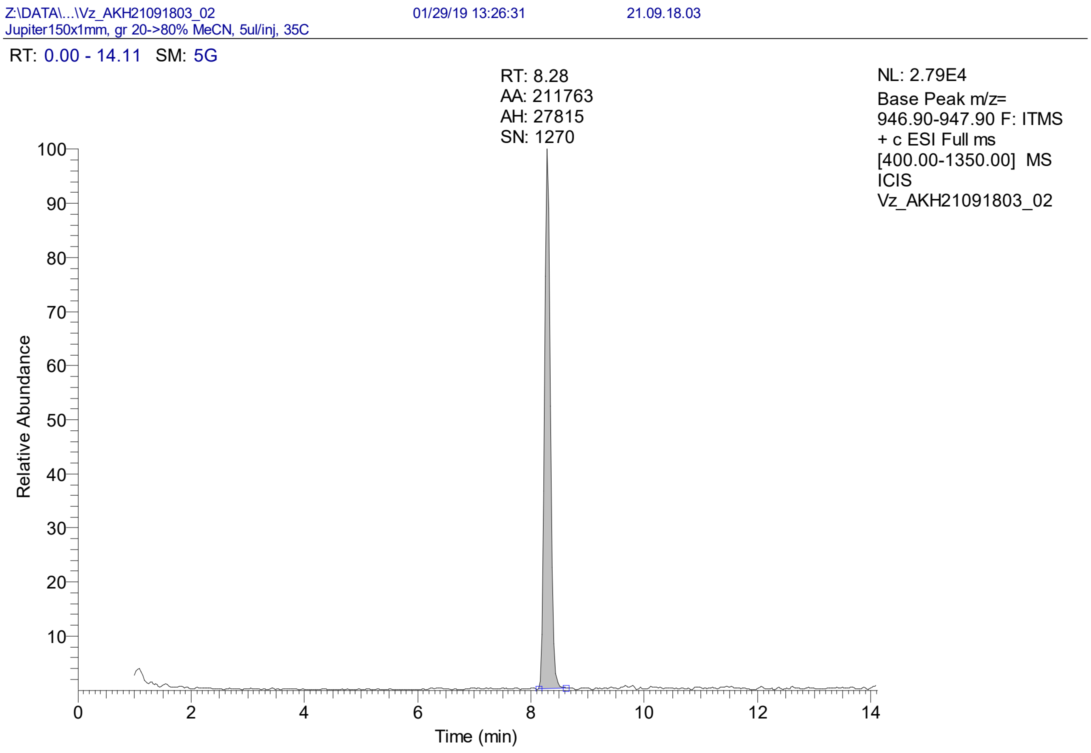

Fig. S1 F. LC-MS chromatogram of the sample, *T. paludosa* corpus cardiacum Peak 2 spiked with the synthetic novel AKH peptide pELTFSPSW-NH_2. The native Peak 2 coelutes with pELTFSPSW-NH_2 which is now code-named Tippa-CC-II.

Fig. S1 G - I. An LC-MS co-elution experiment of the CC extract-derived peptide 3 with MH^+ 917.4 from the crane fly spiked with the synthetic peptide: pELTFSPGW-NH_2; MH^+ = 947.4**.** The extracted chromatograms D - F reveal one prominent peak that co-incides with the retention time of the native peptide, thus indicating that the amino acid in position 2 is Leu, and not Ile. The identity of the third AKH in *T. paludosa* is, therefore, Glomo-AKH.

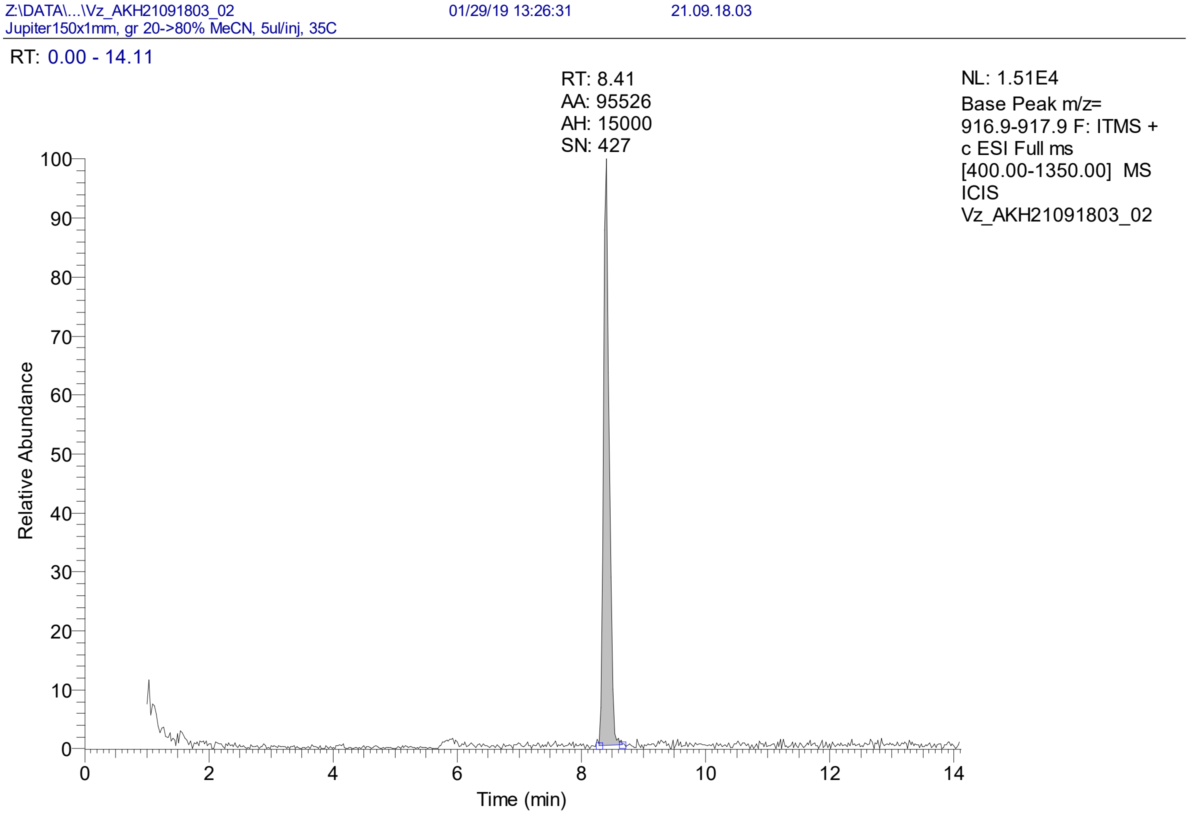

Fig. S1 G. LC-MS chromatogram of the synthetic AKH Glomo-AKH (pELTFSPGW-NH_2) with an extracted mass MH^+ = 917.4.

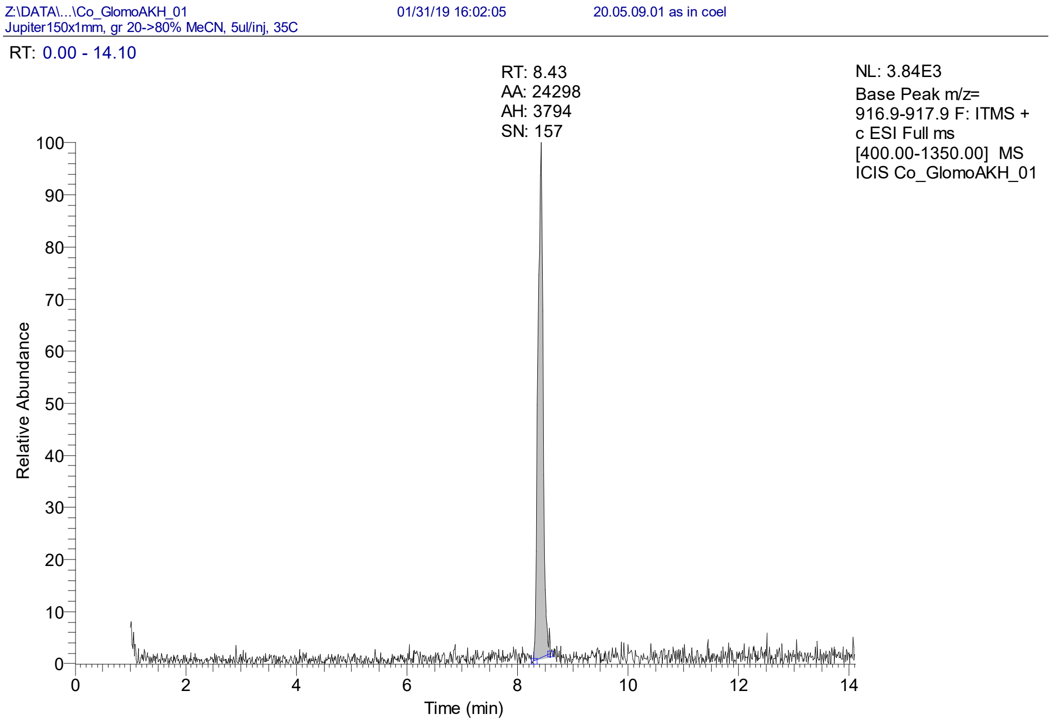

Fig. S1 H. LC-MS chromatogram of the sample, *T. paludosa* corpus cardiacum Peak 3, with an extracted mass MH^+ = 917.4.

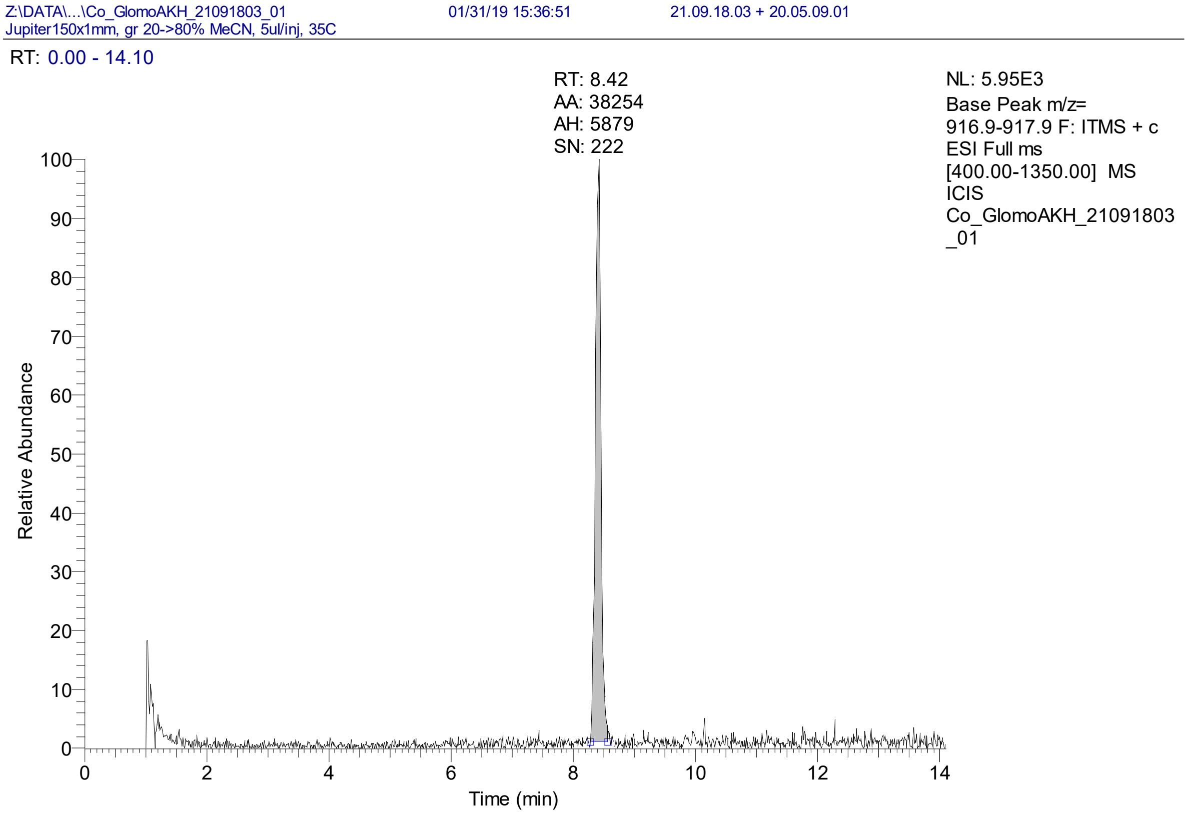

Fig. S1 I. LC-MS chromatogram of the sample, *T. paludosa* corpus cardiacum Peak 3, spiked with the synthetic Glomo-AKH peptide pELTFSPGW-NH_2. The native Peak 3 coelutes with pELTFSPGW-NH_2 = Glomo-AKH.
