# Supplementary material for: The Adipokinetic Peptides in Diptera: Structure, Function, and Evolutionary Trends
Source: Front Endocrinol (Lausanne). 2020 Mar 31;11:153. doi: 10.3389/fendo.2020.00153 (PMC7136388; doi:10.3389/fendo.2020.00153)
Supplement: Supplementary file 2 [file Data_Sheet_2.PDF]

SUPPLEMENTARY FIGURE S2. Sequence elucidation and confirmation of the AKH peptide structure in the black soldier fly *Hermetia illucens* corpus cardiacum extract by HPLC-MS co-elution of the native peak with the corresponding diluted synthetic AKH peptide.

Fig. S2 A - C. Determining the presence and primary amino acid structure of an AKH in a CC extract of *H. Illucens* via LC-MS.

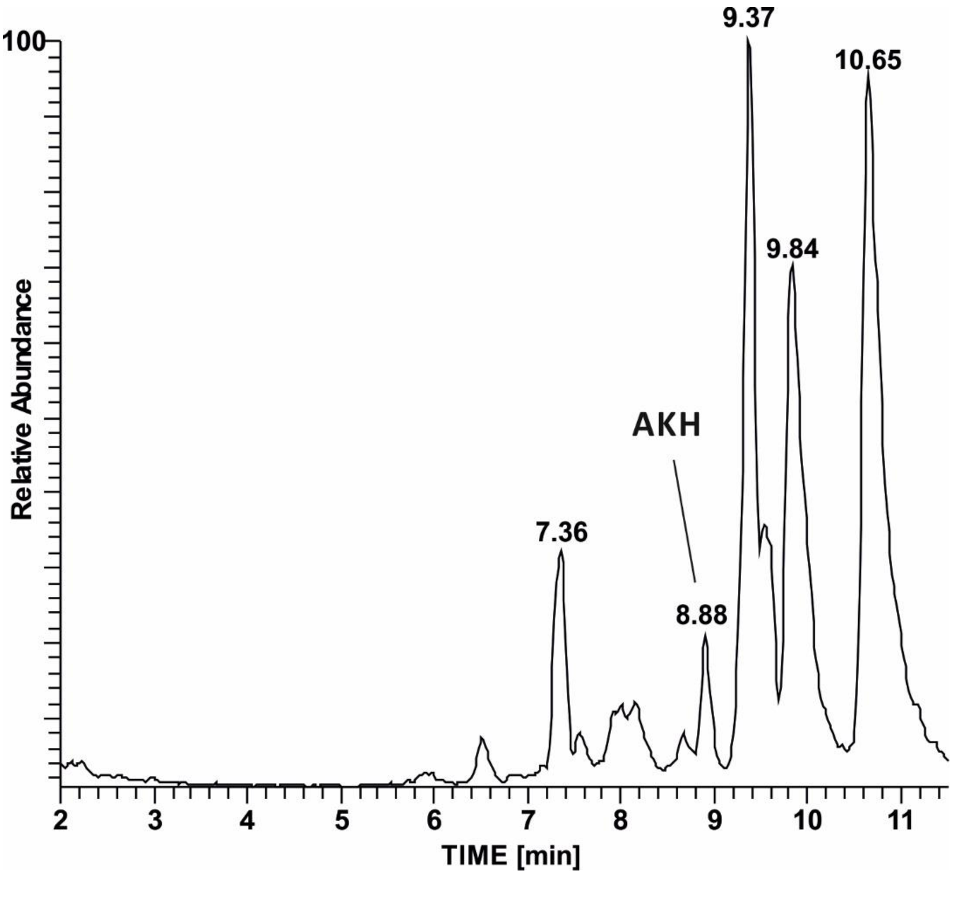

Fig. S2 A. Liquid chromatographic (LC) positive electrospray ionization (+ESI) mass spectrometric (MS) analysis of an extract from corpus cardiacum material of the black soldier fly *Hermetia illucens*. Total ion chromatogram (TIC) obtained by LC-MS analysis detected one AKH eluting at 8.88 min.

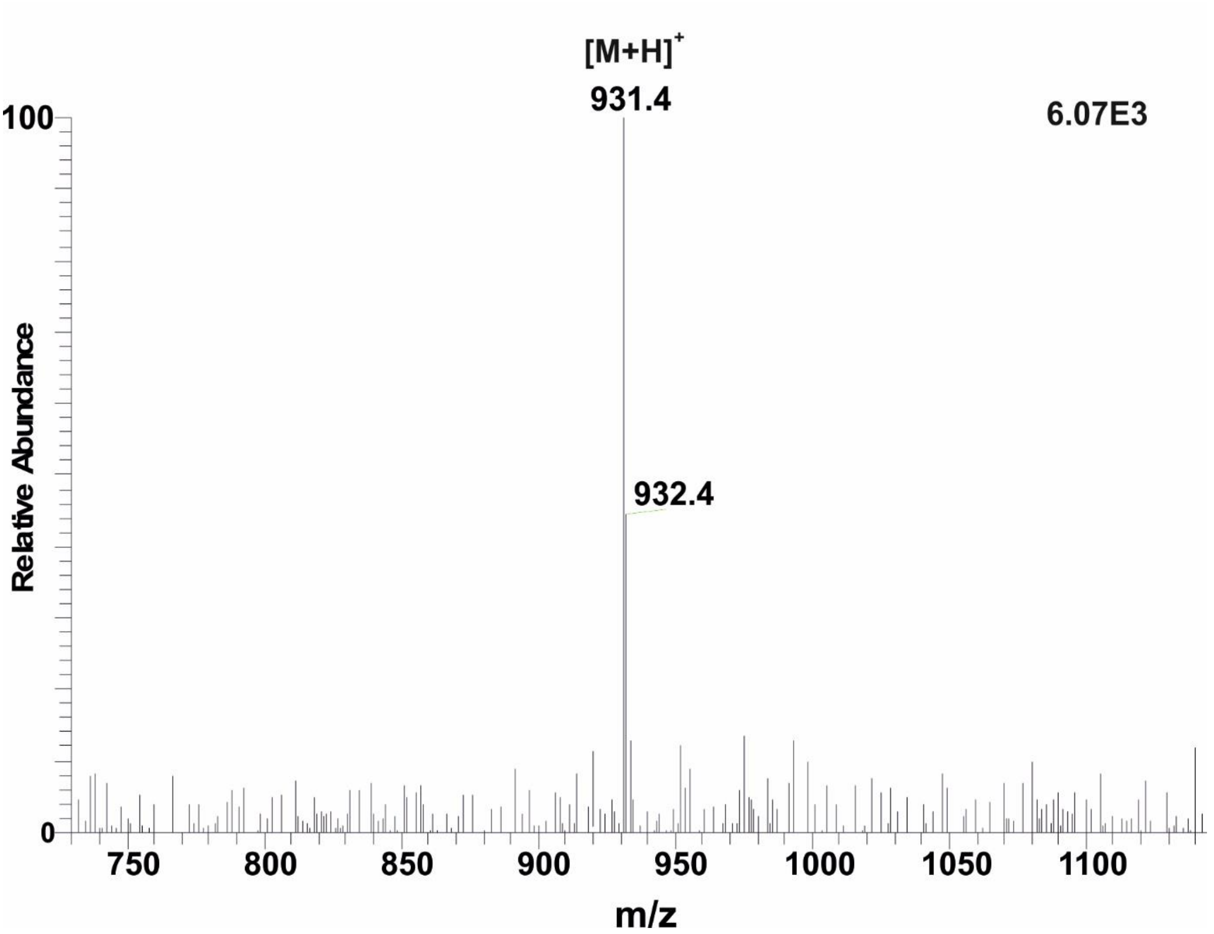

Fig. S2 B. A full scan +ESI mass spectrum recorded from the peak at 8.88 min in (A), showing $[M + H]^+$ at *m/z* 931.4

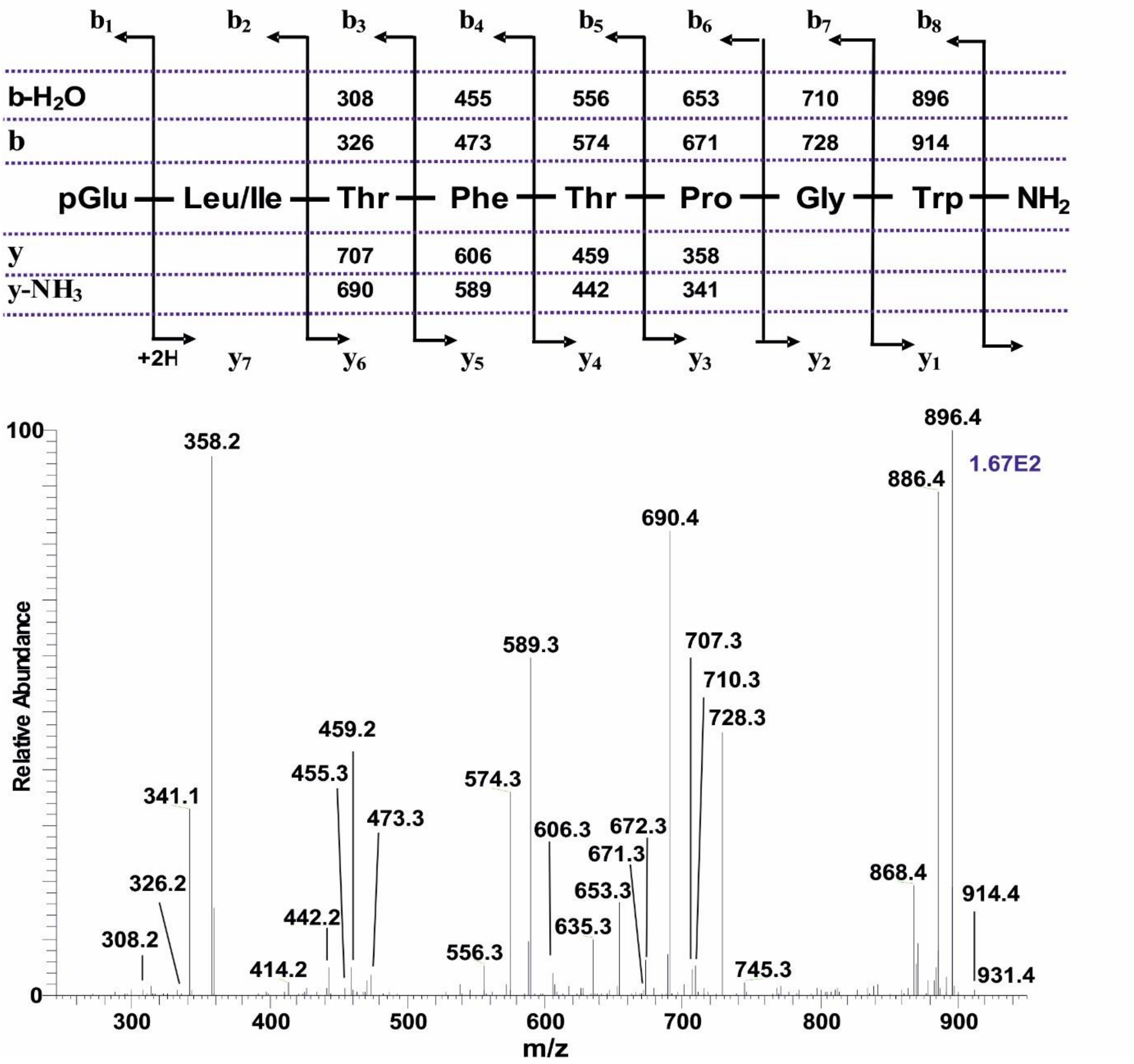

Fig. S2 C. A collision-induced dissociation (CID) tandem MS + ESI spectrum of the ion $[M + H]^+ = 931.4$ in Figure S2 B from the CC of *H. illucens*.

Fig. S2 D - F. Confirmation of the AKH peptide structure of the soldier fly *Hermetia illucens* corpus cardiacum extract by LC-MS co-elution of the native peak with the corresponding synthetic AKH peptide. An extracted ion LC-MS chromatogram is depicted in each case for the detected AKH.

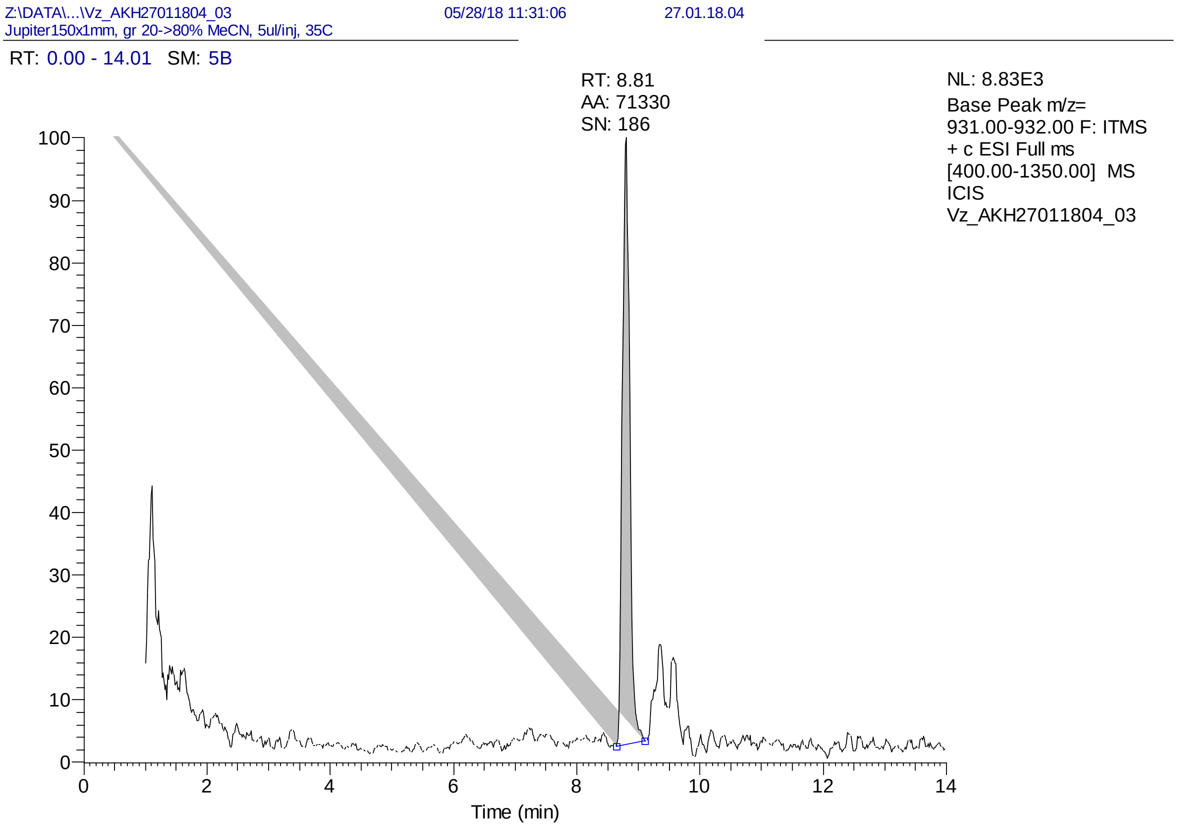

Fig. S2 D. Extracted chromatogram of the peak of *H. illucens* crude CC extract with the mass MH^+ = 931.4.

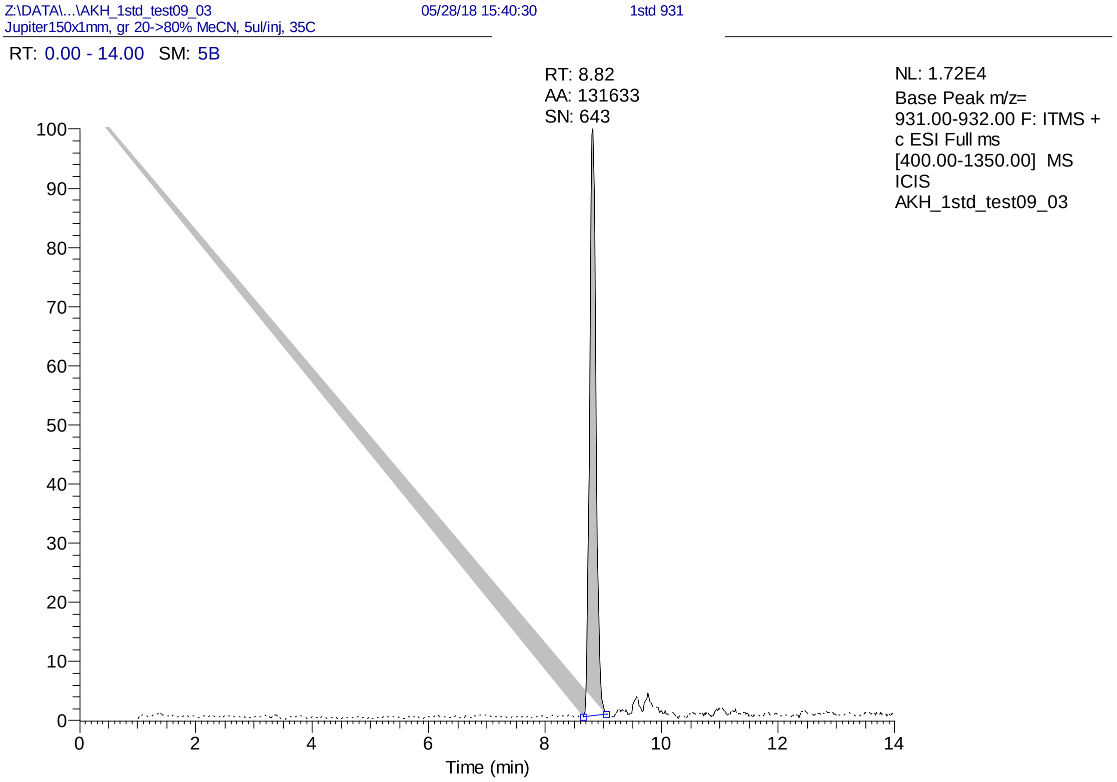

Fig. S2 E. Extracted chromatogram of the peak of synthetic Tabat-AKH (MH^+ = 931.4): pELTFTPGW-NH_2.

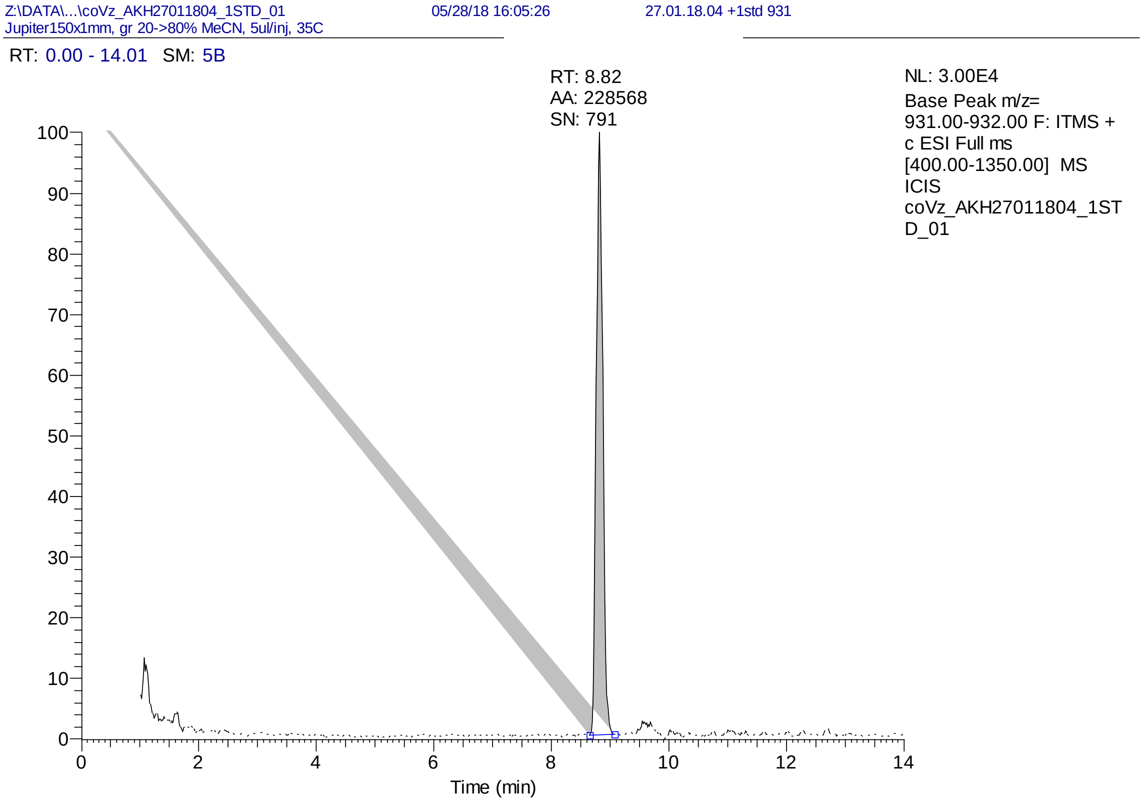

Fig. S2 F. Extracted chromatogram of the peak of crude CC extract with the mass MH^+ = 931.4 spiked with synthetic Tabat-AKH. The single peak proves that the native peptide has Leu at position 2, and is thus, Tabat-AKH.
