# Supplementary material for: The Adipokinetic Peptides in Diptera: Structure, Function, and Evolutionary Trends
Source: Front Endocrinol (Lausanne). 2020 Mar 31;11:153. doi: 10.3389/fendo.2020.00153 (PMC7136388; doi:10.3389/fendo.2020.00153)
Supplement: Supplementary file 3 [file Data_Sheet_3.PDF]

SUPPLEMENTARY FIGURE S3 LC-MS +ESI analysis of an extract from corpus cardiacum material of a mixture of hover fly species of the genus *Eristalis* and confirmation of the four AKH peptide structures by co-elution with synthetic peptides.

Fig. S3 A. Base peak chromatogram of the LC-MS analysis of a crude CC extract from the mixture of hoverfly species, genus *Eristalis*.

Fig. S3 B – E. Extracted LC-MS chromatograms of the mass MH^+ 891.4 (B), 917.4 (C), 975.5 (D) and 1023.5 (E).

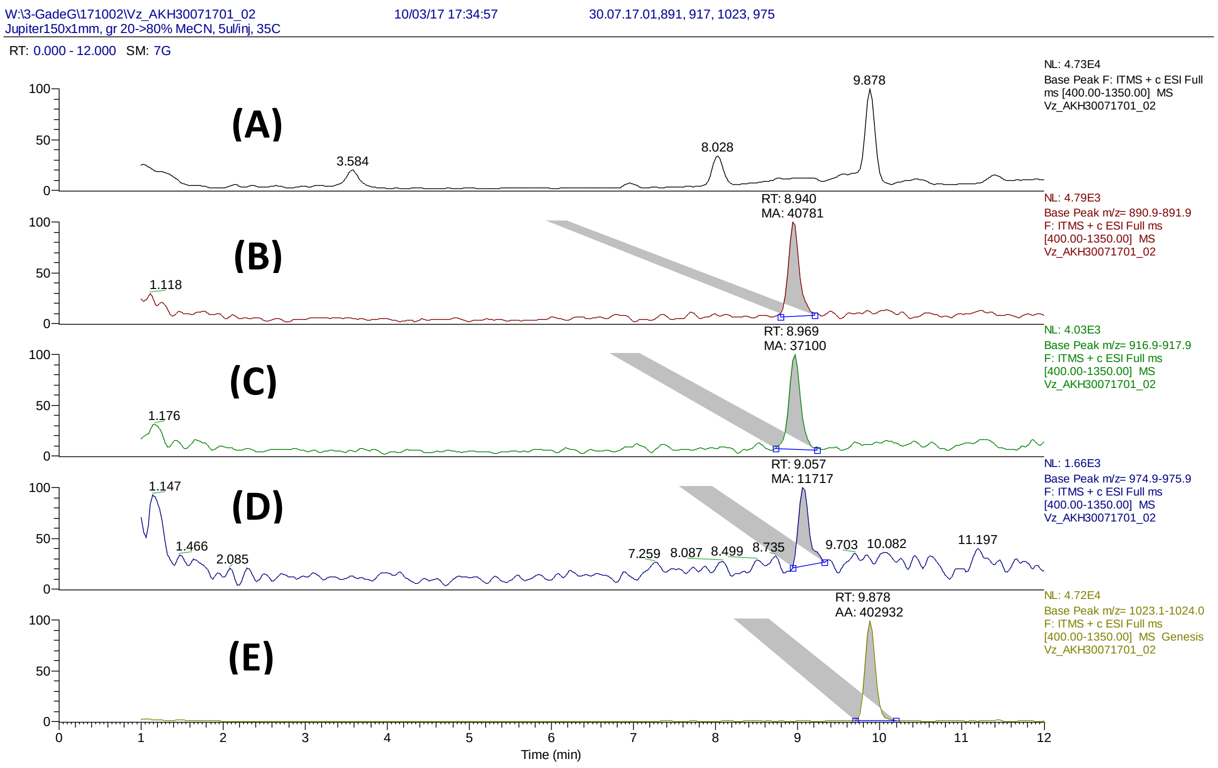

Fig. S3 F – Q. Confirmation of the AKH peptide structures found in the corpus cardiacum extract of the mixture of hoverfly species, genus *Eristalis* by LC-MS co-elution of the native peaks with the corresponding synthetic AKH peptides. An extracted ion LC-MS chromatogram is depicted in each case.

Fig. S3 F – H. Confirmation of the AKH peptide structure with the mass MH^+ = 1023.5 in Fig. S3 E, by LC-MS co-elution of the native peak with the synthetic novel AKH peptide known as Volpe-CC: pELTFSPYW-NH_2. An extracted ion LC-MS chromatogram is depicted in each case.

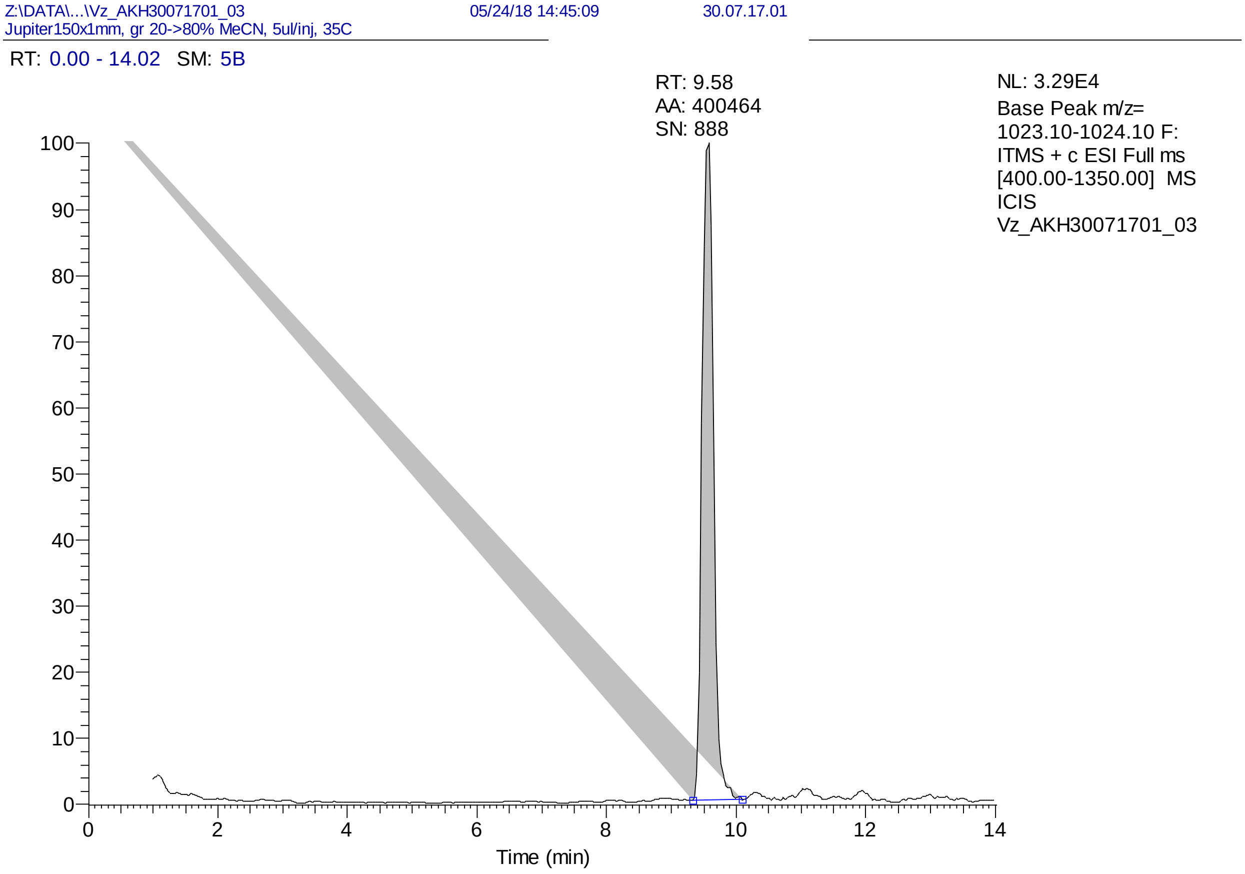

Fig. S3 F. Extracted chromatogram of the peak in Fig. S3 E with the mass MH^+ = 1023.5 from crude CC extract of the mixture of hoverfly species, genus *Eristalis*.

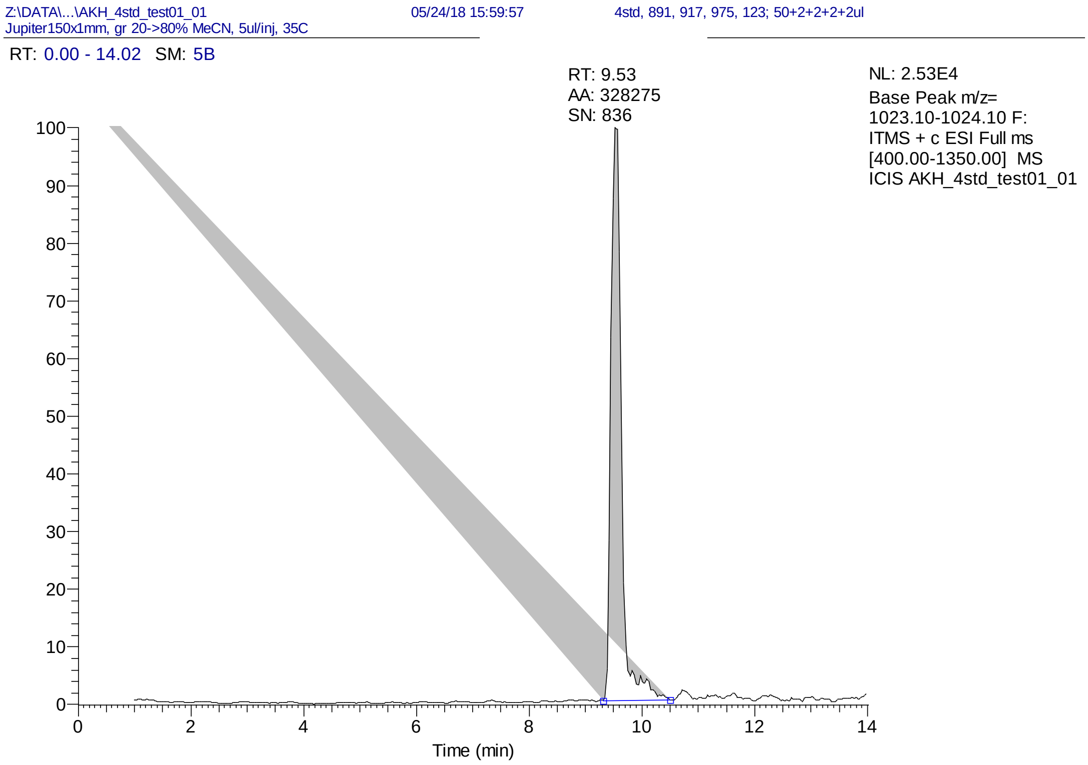

Fig. S3 G. Extracted chromatogram of the peak of the synthetic novel Volpe-CC (MH$^+$ = 1023.5).

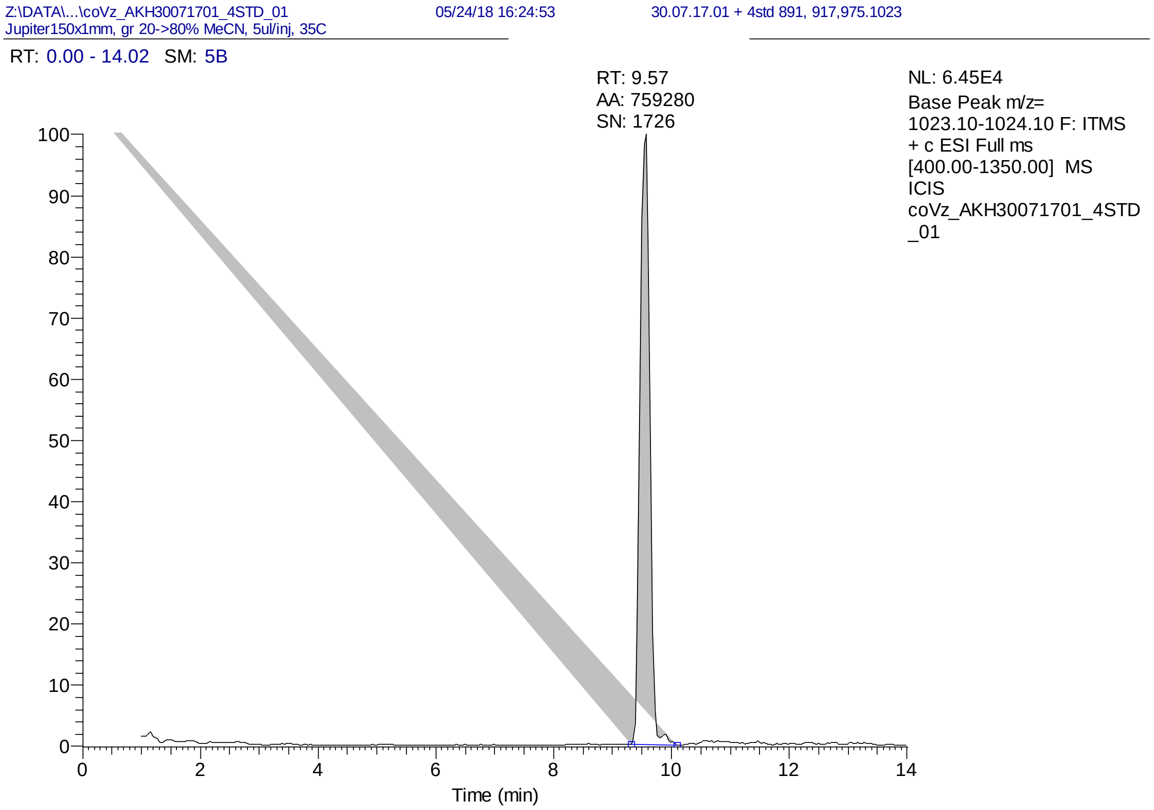

Fig. S3 H. Extracted chromatogram of the peak of crude CC extract with the mass MH$^+$ = 1023.5 spiked with synthetic Volpe-CC. A single peak proves that the native peptide has Leu at position 2 with the structure pELTFSPYW-NH_2.

Fig. S3 I – K. Confirmation of the AKH peptide structure with the mass MH^+ = 975.4 in Fig. S3D, by LC-MS co-elution of the native peak with the synthetic AKH peptide Phote-HrTH: pELTFSPDW-NH_2. An extracted ion LC-MS chromatogram is depicted in each case.

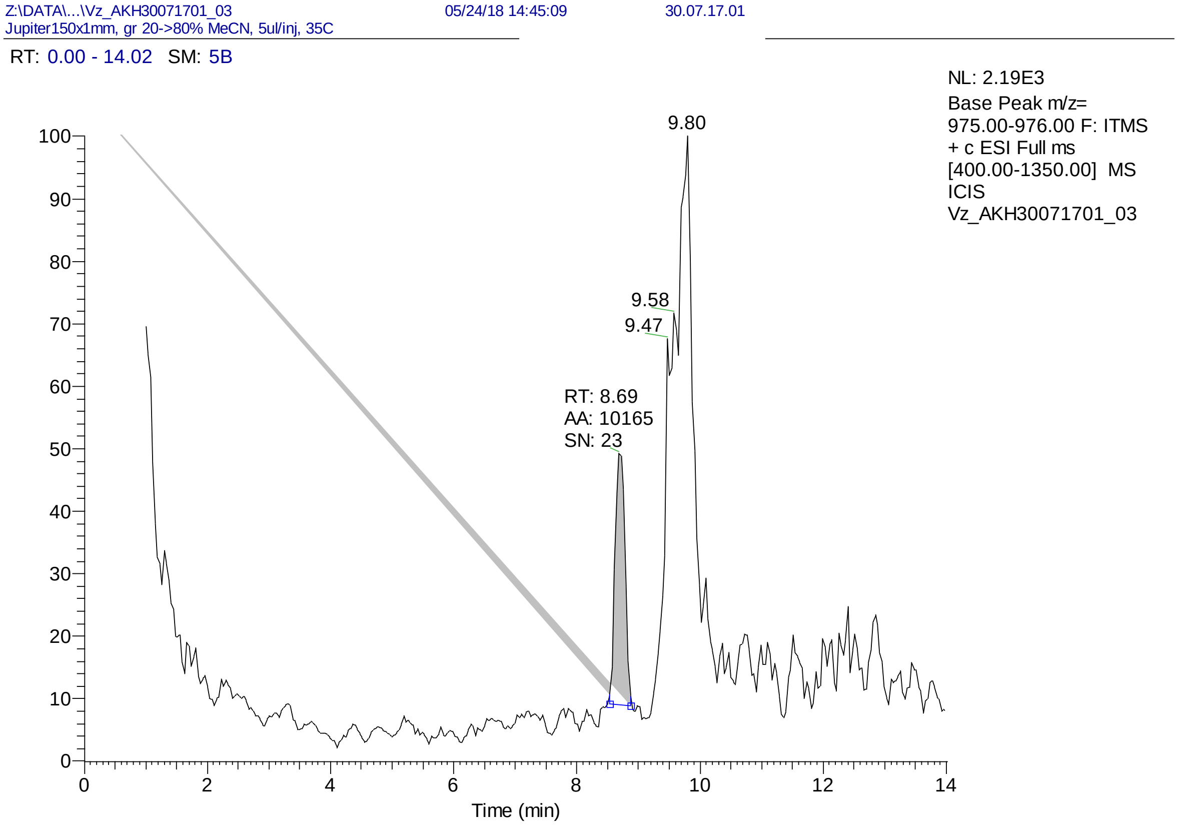

Fig. S3 I. Extracted chromatogram of the peak in Fig. S3D with the mass MH^+ = 975.4 from crude CC extract of the mixture of hoverfly species, genus *Eristalis*.

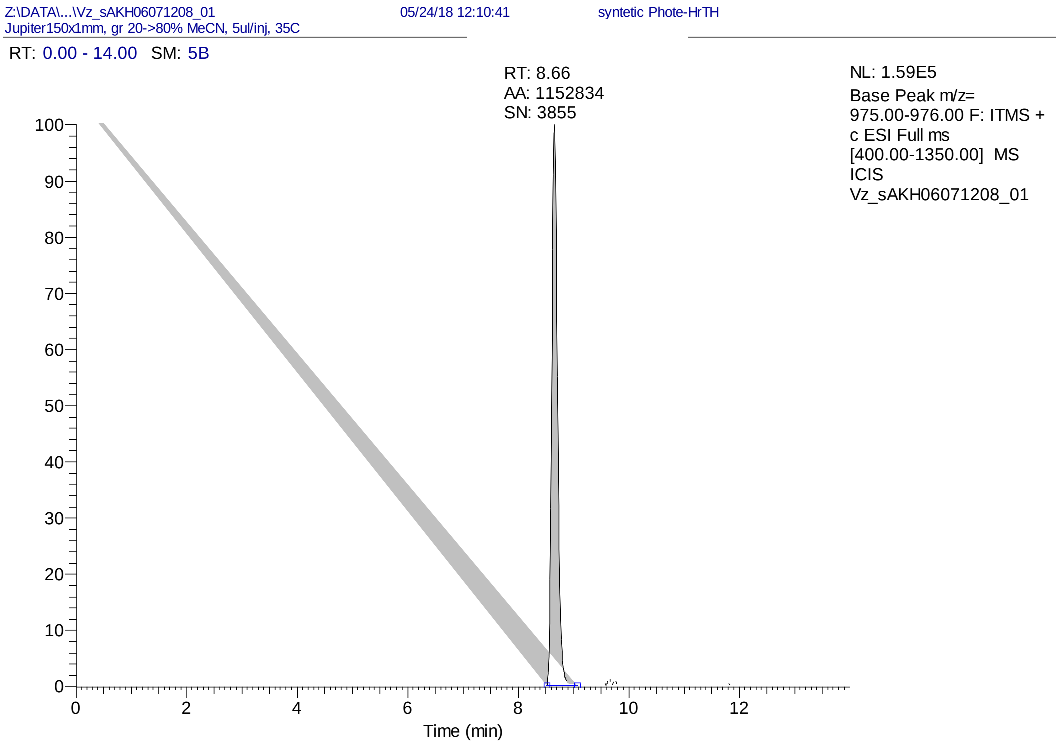

Fig. S3 J. Extracted chromatogram of the peak of synthetic Phote-HrTH (MH^+ = 975.4)

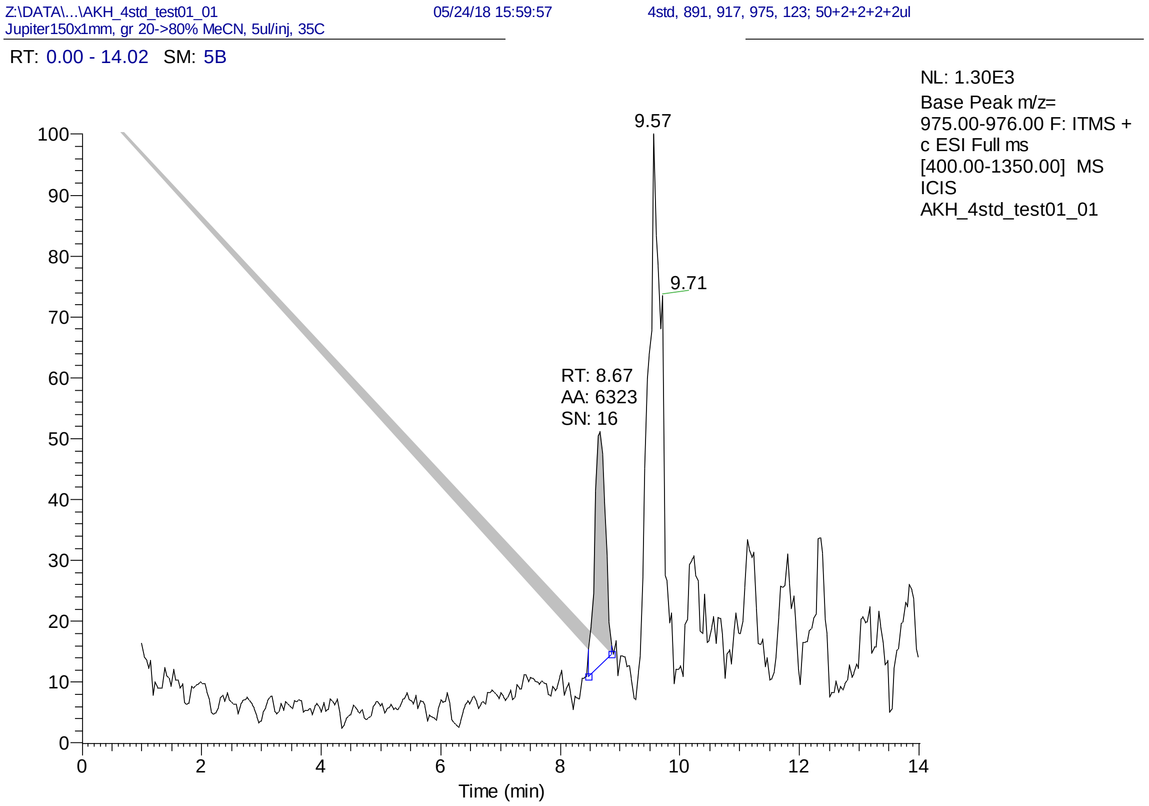

Fig. S3 K. Extracted chromatogram of the peak of crude CC extract with the mass MH+ = 975.4 spiked with synthetic Phote-HrTH. The co-elution peak proves that the native peptide has Leu at position 2 with the structure pELTFSPDW-NH_2.

Fig. S3 L – N. Confirmation of the AKH peptide structure with the mass MH^+ = 917.44 in Fig. S3C, by LC-MS co-elution of the native peak with the synthetic AKH peptide Glomo-AKH: pELTFSPGW-NH_2. An extracted ion LC-MS chromatogram is depicted in each case.

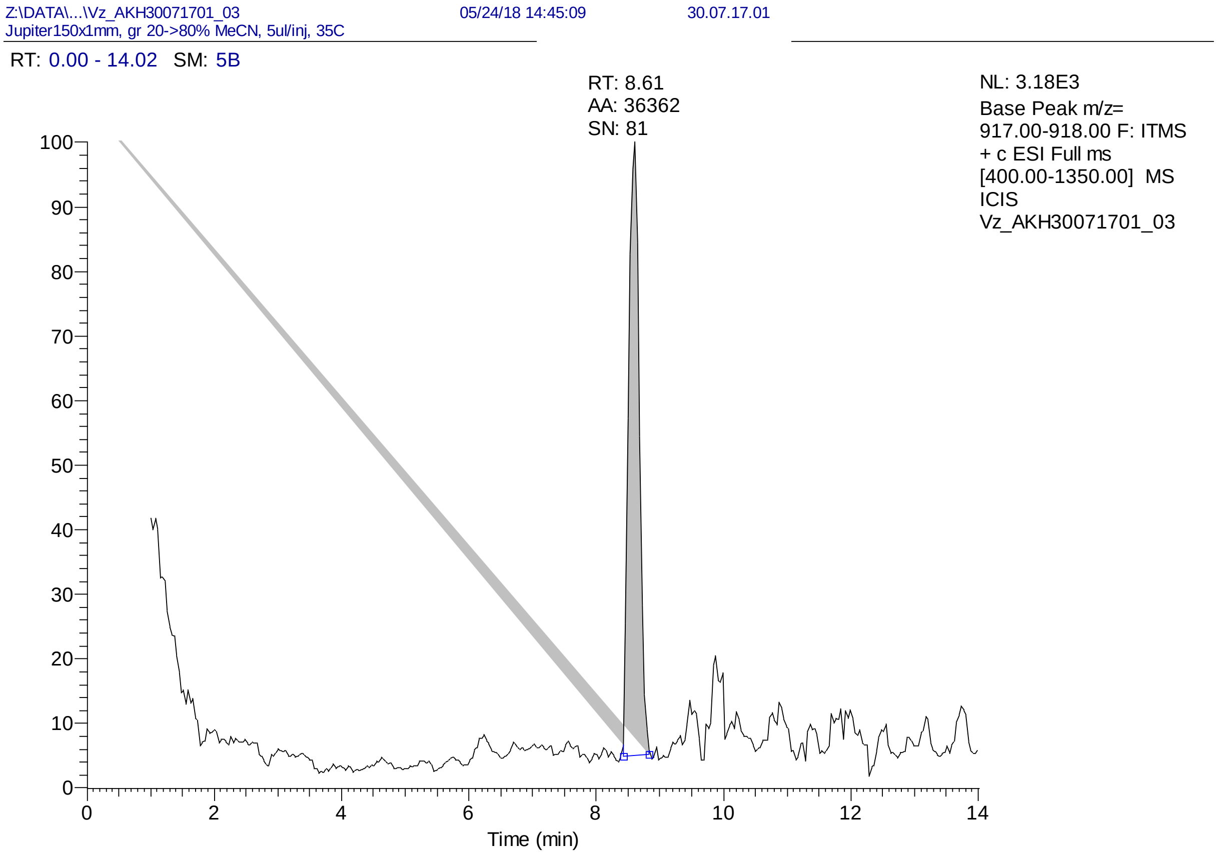

Fig. S3 L. Extracted chromatogram of the peak in Fig. S3C with the mass MH^+ = 917.4 from crude CC extract of the mixture of hoverfly species, genus *Eristalis*.

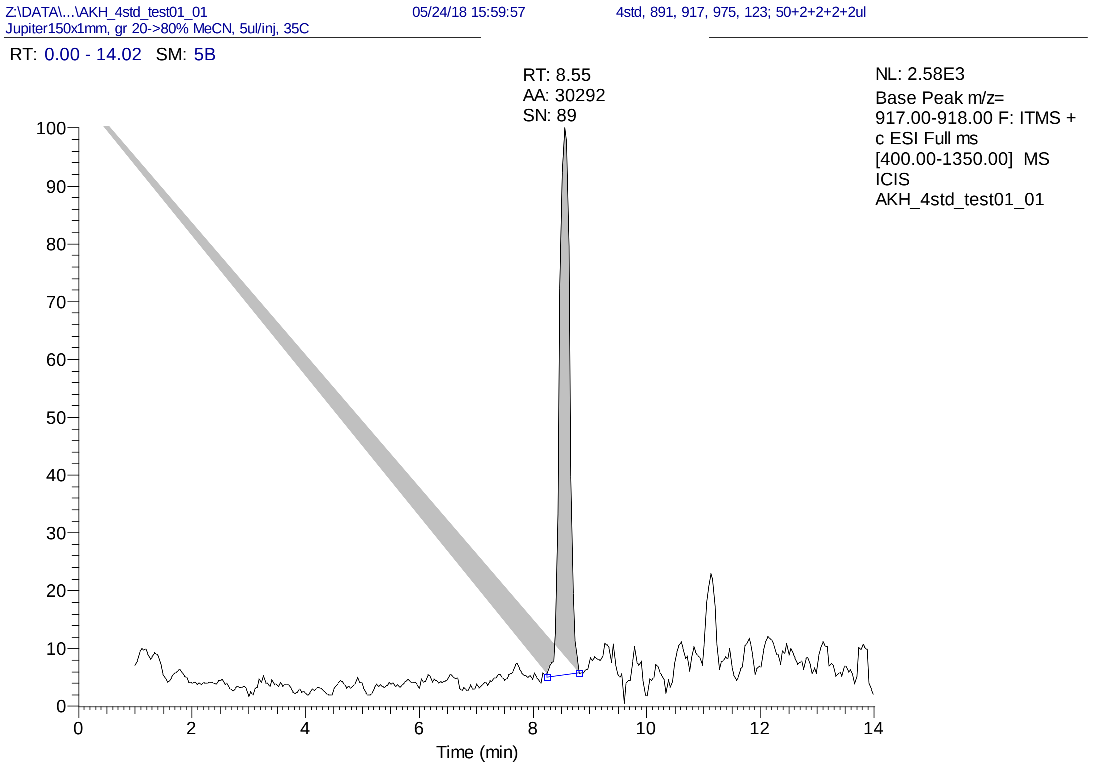

Fig. S3 M. Extracted chromatogram of the peak of synthetic Glomo-AKH ($MH^+ = 917.4$).

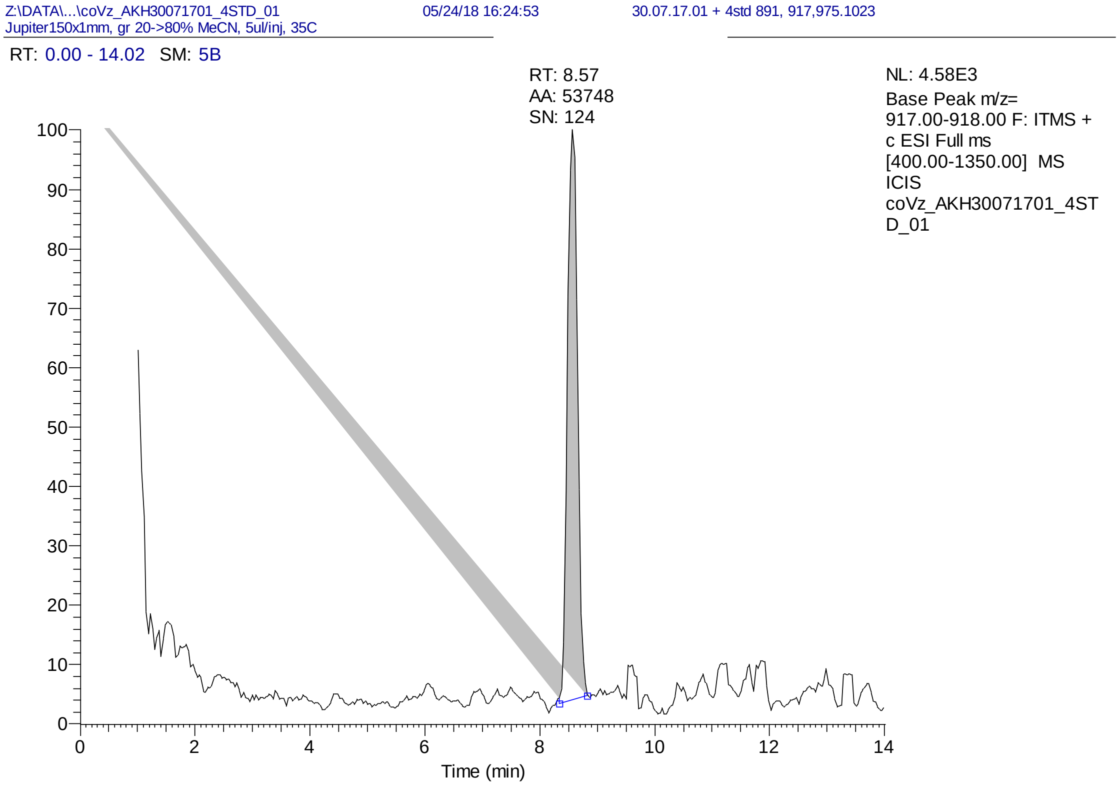

Fig. S3 N. Extracted chromatogram of the peak of crude CC extract with the mass $MH^+ = 917.4$ spiked with synthetic Glomo-AKH. The co-elution peak proves that the native peptide has Leu at position 2 with the structure pELTFSPGW-NH_2.

Fig. S3 O – Q. Confirmation of the AKH peptide structure with the mass MH^+ = 891.4 in Fig. S3B, by LC-MS co-elution of the native peak with the synthetic novel AKH peptide Eriss-CC: pELTFSAGW-NH_2. An extracted ion LC-MS chromatogram is depicted in each case.

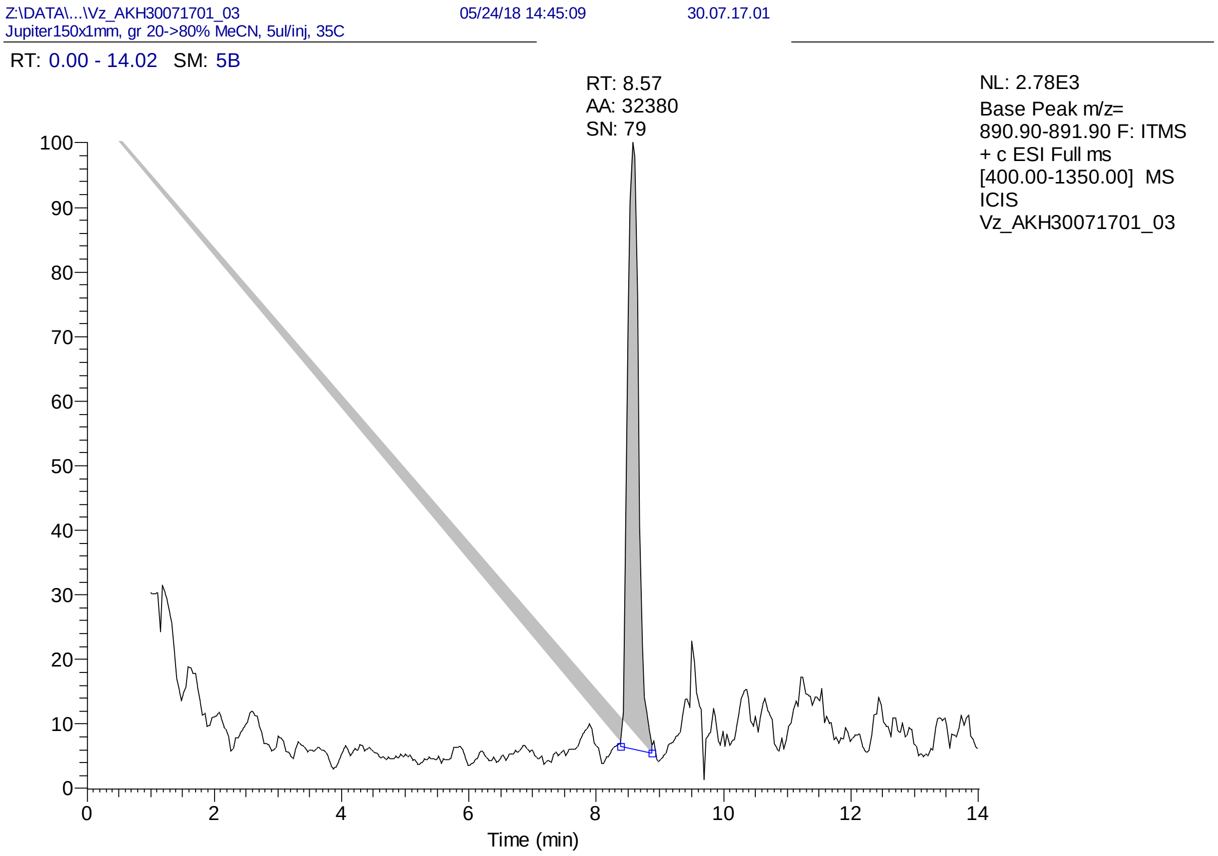

Fig. S3 O. Extracted chromatogram of the peak in Fig. S3B with the mass MH^+ = 891.4 from crude CC extract of the mixture of hoverfly species, genus *Eristalis*.

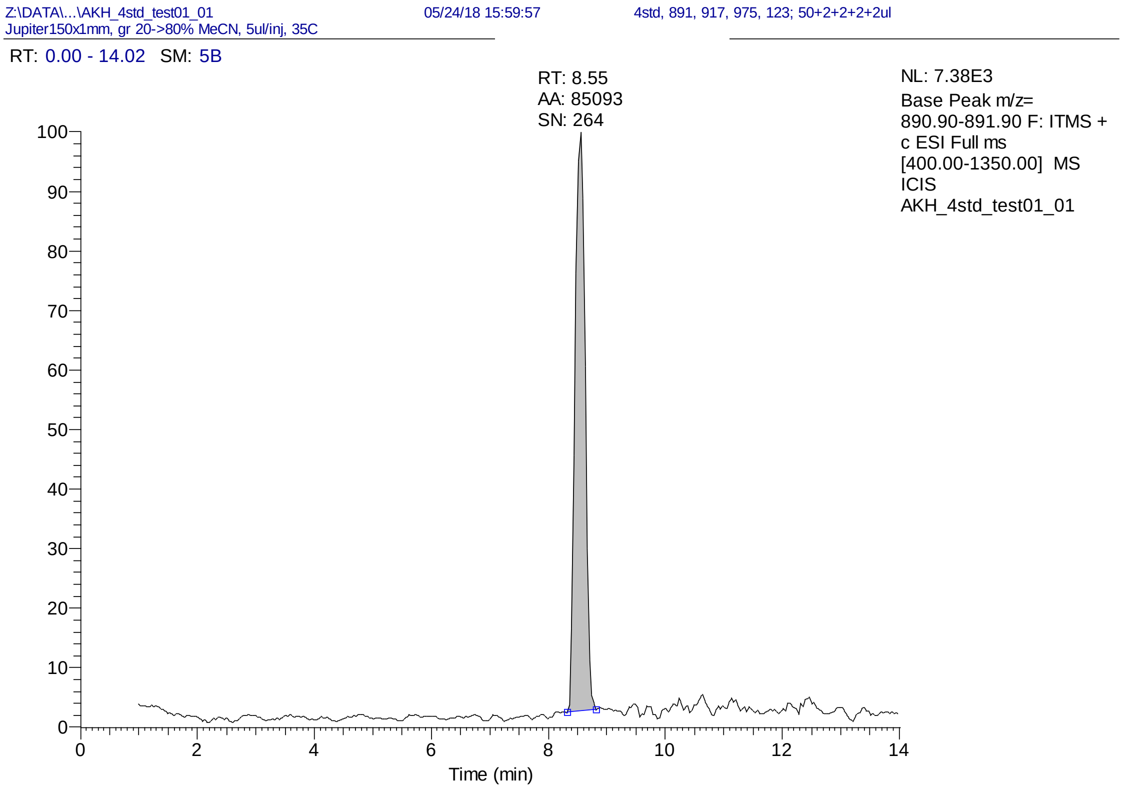

Fig. S3 P. Extracted chromatogram of the peak of the synthetic novel AKH (MH^+ = 891.4): pELTFSAGW-NH_2.

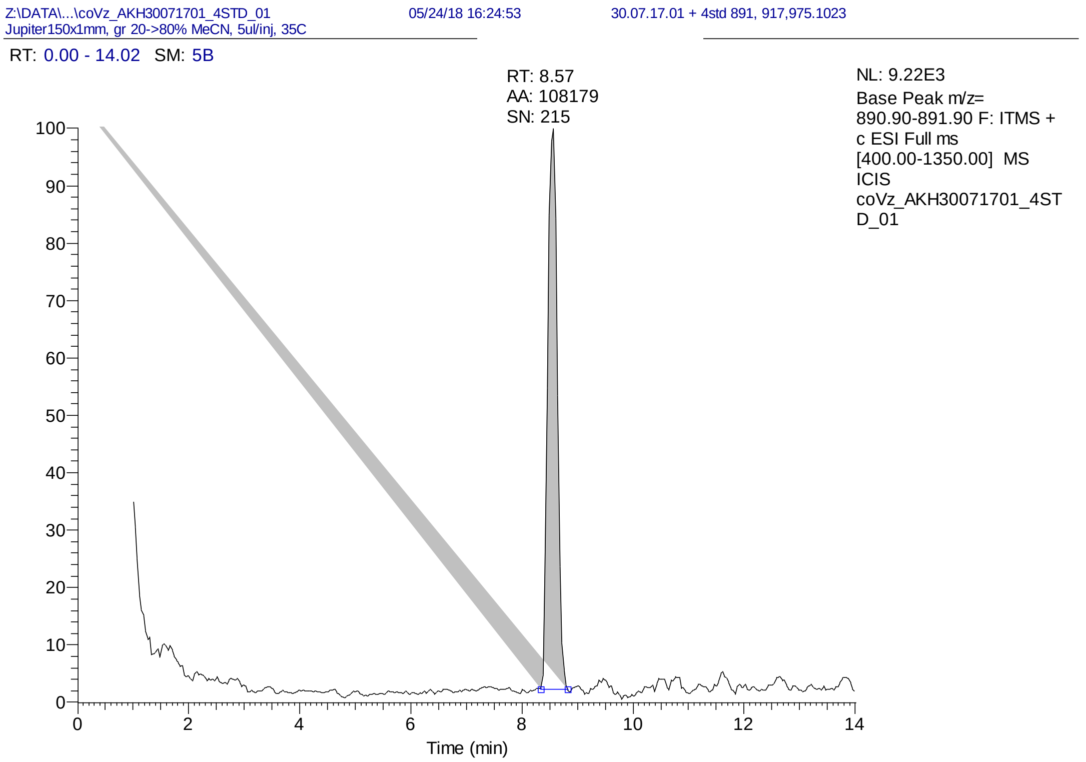

Fig. S3 Q. Extracted chromatogram of the peak of crude CC extract with the mass MH^+ = 891.4 spiked with a synthetic novel-AKH: pELTFSAGW-NH_2. The co-elution peak proves that the native peptide shares the structure of this novel AKH, now code-named Eriss-CC.
