# Supplementary material for: The Adipokinetic Peptides in Diptera: Structure, Function, and Evolutionary Trends
Source: Front Endocrinol (Lausanne). 2020 Mar 31;11:153. doi: 10.3389/fendo.2020.00153 (PMC7136388; doi:10.3389/fendo.2020.00153)
Supplement: Supplementary file 4 [file Data_Sheet_4.PDF]

SUPPLEMENTARY FIGURE S4. LC-MS +ESI analysis of an extract from corpus cardiacum material of the fruit fly C*eratitis capitata* and confirmation of the AKH peptide structure by co-elution with synthetic peptide

Fig. S4 A - C. Determining the presence and primary amino acid structure of an AKH in a CC extract of C. *capitata* via LC-MS.

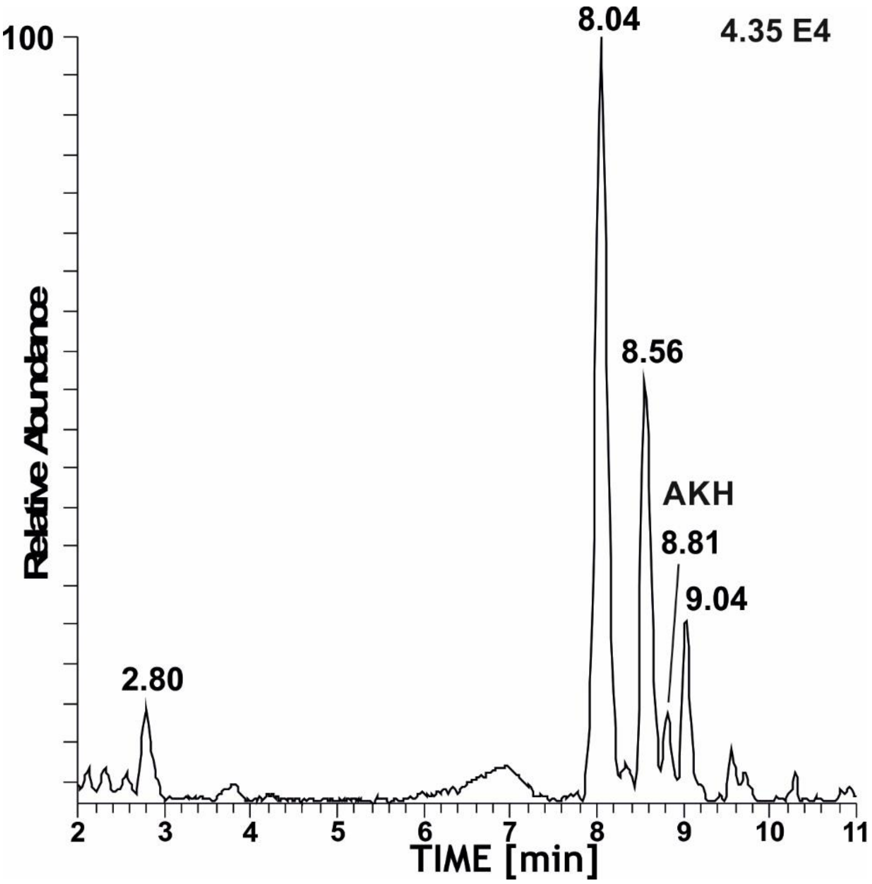

Fig. S4 A. A total ion chromatogram showing the detection of an adipokinetic hormone at 8.81 min.

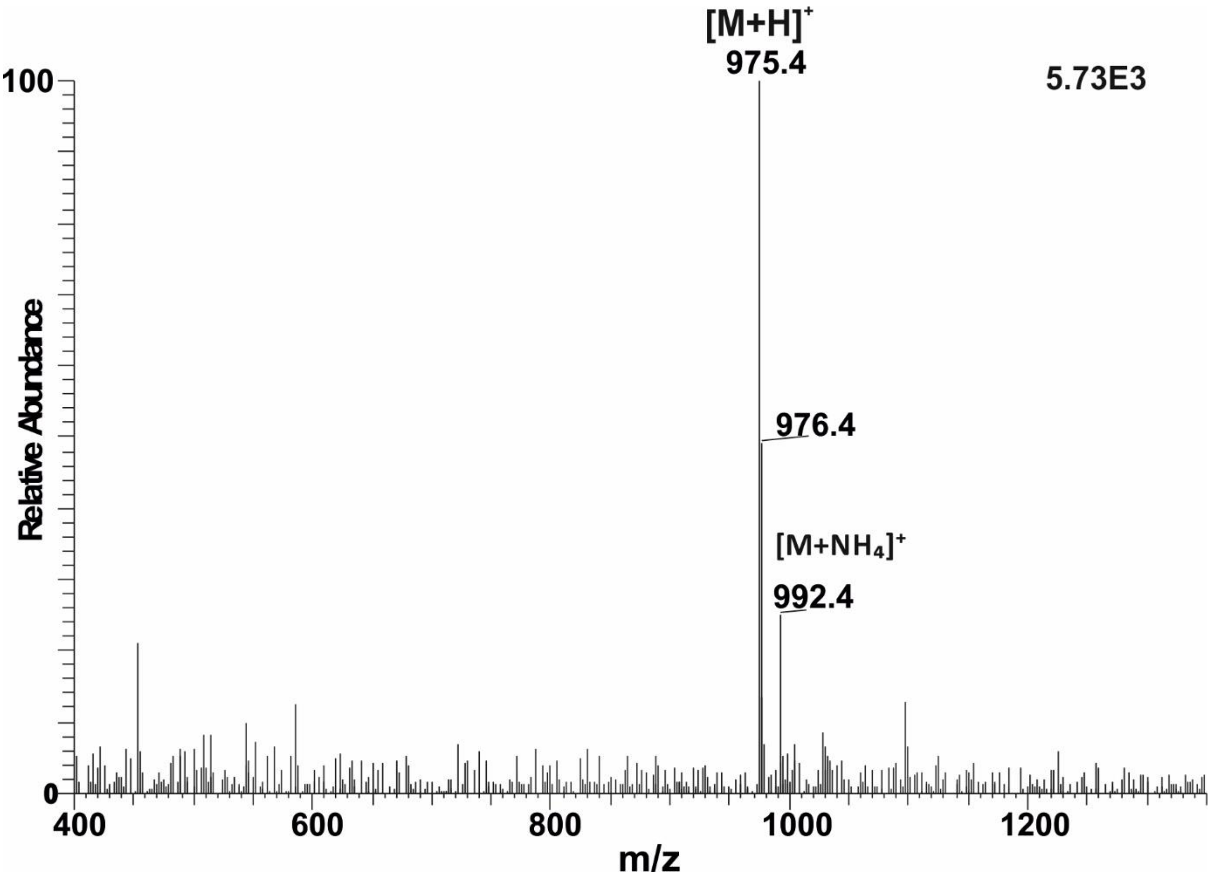

Fig. S4 B. A full scan +ESI mass spectrum recording of the peak at 8.81 min in Fig. S4A showing $[M + H]^+$ at *m*/*z* 975.4.

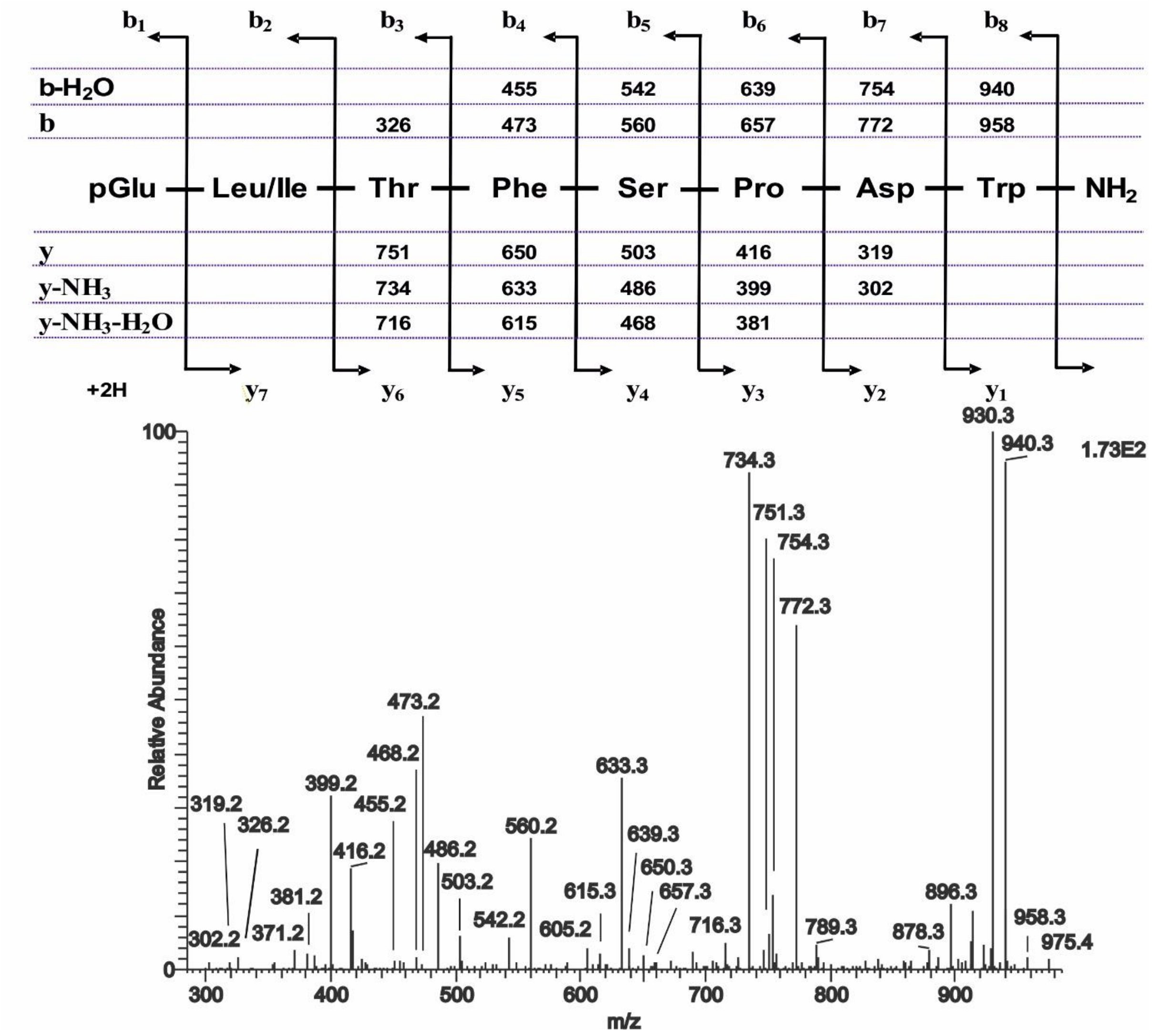

Fig. S4 C. A CID tandem + ESI mass spectrum of the ion $[M + H]^+$ = 975.4 in Fig. S4B from the CC of the fruit fly C. *capitata*. The inset shows the proposed peptide sequence together with the b- and y-type diagnostic fragment ions observed in the MS^2 spectrum.

Fig. S4 D - F. Confirmation of the AKH peptide structure of the fruit fly C*eratitis capitata* corpus cardiacum extract by LC-MS co-elution of the native peak with the corresponding synthetic AKH peptide. An extracted ion LC-MS chromatogram is depicted in each case for the detected AKH.

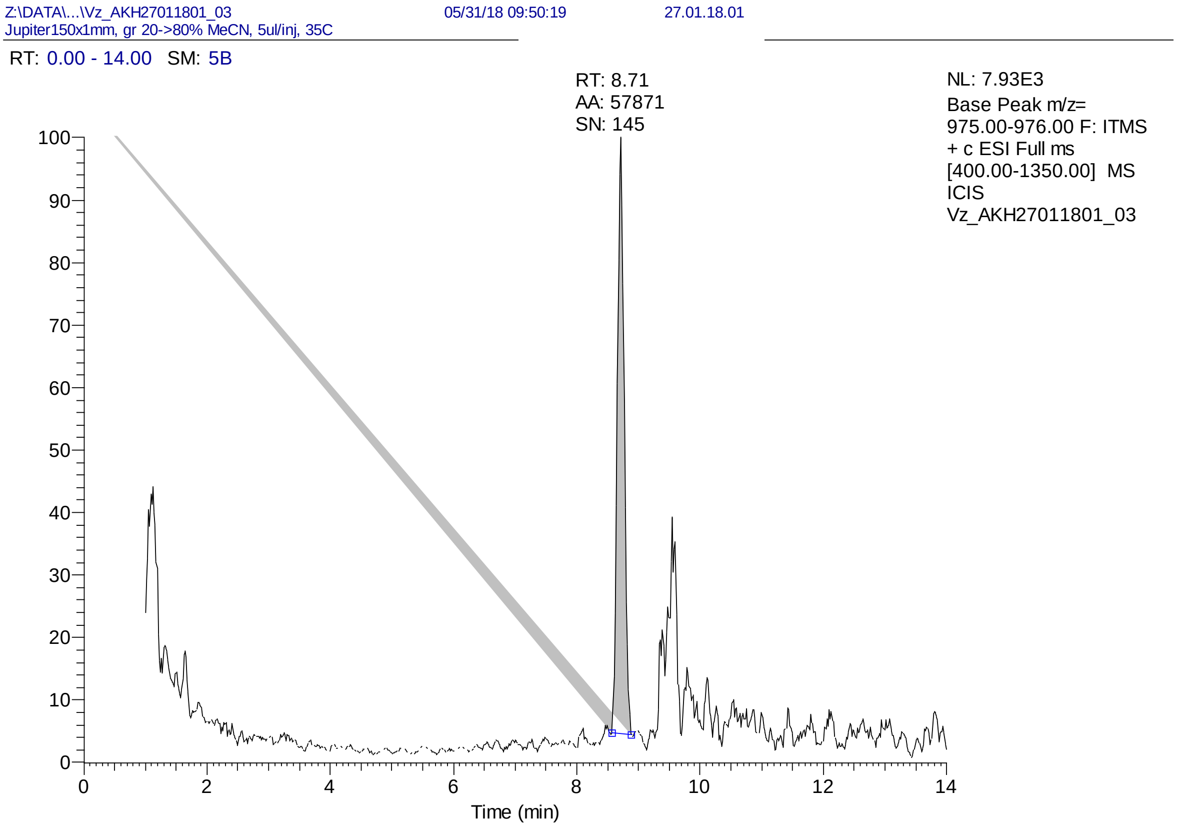

Fig. S4 D. Extracted chromatogram of the peak of C. *capitata* crude CC extract with the mass MH+ = 975.4.

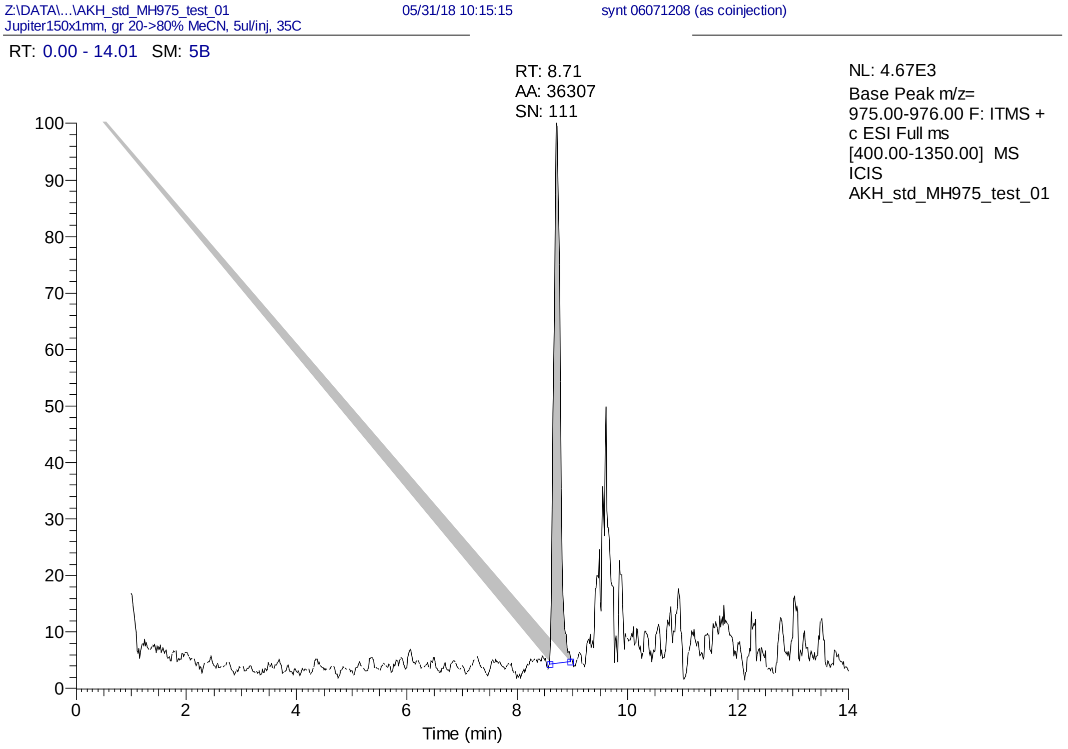

Fig. S4 E. Extracted chromatogram of the peak of synthetic Phote-HrTH (MH^+ = 975.5).

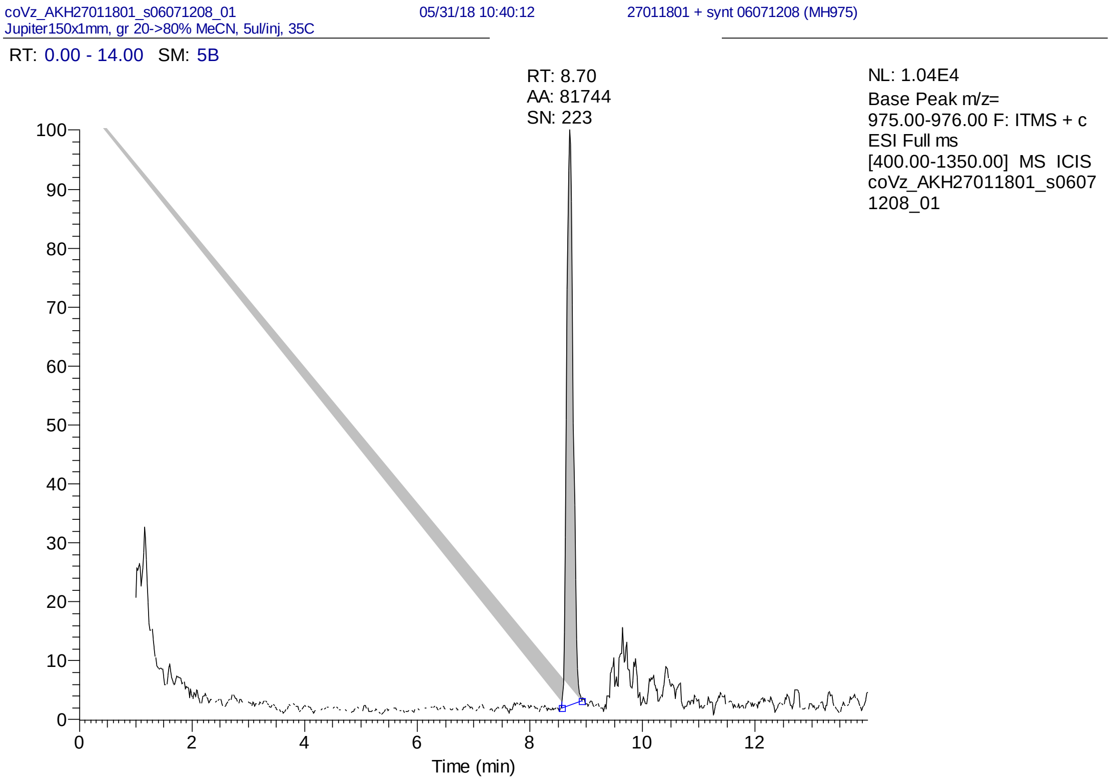

Fig. S4 F. Extracted chromatogram of the peak of crude CC extract with the mass MH^+ = 975.4 spiked with synthetic Phote-HrTH. The single peak proves that the native peptide has Leu at position 2, and is thus, Phote-HrTH: pELTFSPDW-NH_2.
